# From genome to lifestyle: adaptive strategies of *Pseudomonas* sp. AU10 and its Antarctic lineage

**DOI:** 10.1128/aem.02470-25

**Published:** 2026-03-24

**Authors:** César X. García-Laviña, Pablo Smircich, Susana Castro-Sowinski

**Affiliations:** 1 Sección Bioquímica, Facultad de Ciencias, Universidad de la República 56724 , Montevideo, Uruguay; 2 Sección Genómica Funcional, Facultad de Ciencias, Universidad de la República 56724 , Montevideo, Uruguay; 3 Departamento de Genómica, Instituto de Investigaciones Biológicas Clemente Estable, Ministerio de Educación y Cultura 113067, Montevideo, Uruguay; University of Milano-Bicocca, Milan, Italy

**Keywords:** Antarctic bacteria, extremophiles, bacterial evolution, comparative genomics, metabolic reconstruction

## Abstract

Antarctica harbors diverse *Pseudomonas* lineages, yet their evolutionary histories and ecological strategies remain poorly understood. Growing genomic resources now enable comparative analyses that link genome information to lifestyles. Here, we report the complete genome of *Pseudomonas* sp. AU10, an Antarctic freshwater isolate, and use it as a model to investigate polar adaptation through phylogenomics, pangenomics, metabolic reconstruction, and phenotypic assays. Incorporating the 40 most closely related genomes from public databases, we identified that all 11 strains from permanently cold environments clustered into two well-defined monophyletic clades with contrasting biogeographic patterns: a geographically restricted “Antarctic clade,” to which AU10 belongs, and a “cold clade” distributed worldwide from the Himalayas to the Arctic and Antarctica. The two lineages lacked a shared conserved gene set for adaptation to permanently frozen habitats; instead, clade-specific genomic signatures suggested independent adaptive strategies. The Antarctic clade resolved into two species, both sharing distinctive features related to ionic/osmotic homeostasis, respiratory flexibility, and virulence factors, including insecticidal toxins. Within this clade, AU10’s species showed expanded virulence factors and iron acquisition systems. Genomic evidence pointed to a potential pathogenic lifestyle involving freshwater arthropods, supported by AU10’s metabolism oriented toward protein, lipid, and chitin hydrolysis and the overrepresentation of transport systems for amino and organic acids. Intraspecific variability involved the replacement of O-antigen gene clusters and a mobilome comprising a natural plasmid and several prophages carrying UV-resistance and outer-envelope modification genes. Overall, these findings place AU10 within a long-evolving Antarctic lineage with distinct adaptations, offering insights into bacterial evolution in extreme environments.

**IMPORTANCE:**

Antarctica is one of the most extreme environments on Earth, yet it sustains diverse microbial life. How these microorganisms adapt to permanent cold conditions and the lifestyles they adopt remain difficult to elucidate. The growing wealth of genomic data offers opportunities to reconstruct these evolutionary histories. Still, in versatile genera such as *Pseudomonas*, extracting consistent biological meaning from comparative genomics remains challenging. Here, we focused on a restricted set of genomes closely related to the Antarctic strain *Pseudomonas* sp. AU10 and integrated genomic analyses with phenotypic assays and metabolic reconstruction. This strategy uncovered two cold-adapted lineages with contrasting biogeographic patterns, highlighted lineage-specific genomic features associated with adaptation to the extreme Antarctic environment, and provided insights into their ecological strategies. Ultimately, this approach underscores the value of linking genomic, phenotypic, and ecological information to illuminate bacterial evolution in extreme environments.

## INTRODUCTION

The genus *Pseudomonas* comprises a highly diverse group of Gram-negative bacteria within the class Gammaproteobacteria, renowned for their metabolic versatility, adaptability to environmental stress, and wide range of ecological strategies (1). Members of this genus inhabit virtually all terrestrial and aquatic ecosystems, from soils and freshwater bodies to marine environments, and display lifestyles ranging from innocuous environmental saprobes to plant-growth promoters, phytopathogens, entomopathogens, and opportunistic pathogens of mammals, including humans (1–3). In Antarctica, one of the most inhospitable regions on Earth, records of *Pseudomonas* date back to the 1970s (4, 5), but systematic study of these native dwellers began only two decades ago, resulting in the description and genome sequencing of more than a dozen novel species isolated from soils, rocks, ponds, marine sediments, microbial mats, and fauna (6–13). It can be presumed that these native species have undergone a long evolutionary history in Antarctica, leading to specific adaptations to its harsh conditions, such as low temperatures, frequent freeze–thaw cycles, and intense ultraviolet radiation (14).

Despite increasing efforts to unveil the diversity of Antarctic *Pseudomonas*, their ecology and lifestyle strategies in natural habitats remain poorly understood (15, 16). In contrast, several studies have begun to elucidate the molecular bases that enable members of the genus to thrive as cold-adapted extremophiles (17–20). Advances in high-throughput sequencing have enabled comparative genomic approaches to address such questions, particularly through phylogenomic and pangenomic analyses. These methods clarify the relationships among closely related strains and capture the core and accessory genome components of the group. The resulting patterns reflect lineage-specific gene gain via horizontal transfer and gene loss, processes recognized as major drivers of bacterial evolution (21, 22). Thus, they provide insights into the distinct environmental conditions and selective pressures each lineage has faced over evolutionary time (2). Such genome-to-lifestyle links were first demonstrated in intracellular pathogens, such as *Buchnera aphidicola* and *Mycobacterium leprae*, where massive genome reduction was tied to obligate, host-dependent lifestyles (23, 24). They were later extended to environmental bacteria, where comparative genome analyses have provided valuable insights into environmental adaptation across diverse taxa and ecological contexts, including marine bacteria (25, 26), polar microorganisms (27–29), and diverse lineages of *Pseudomonas* (2, 3, 30).

In this study, we generated and analyzed the complete genome sequence of *Pseudomonas* sp. AU10, a freshwater Antarctic isolate recognized for producing cold-active enzymes of biotechnological interest (31–33) and previously shown to be psychrotolerant and resistant to several heavy metals, metalloids, and antibiotics (34, 35). Using AU10’s genome as reference, we performed phylogenomic and pangenomic analyses with its closest relatives retrieved from public databases to clarify its taxonomic placement and explore patterns of diversification and adaptation. This framework positioned AU10 within a long-evolving Antarctic clade and revealed lineage-specific gene gains and losses associated with ecological specialization. By integrating genomic, phenotypic, and biochemical evidence, we provide insights into AU10’s evolutionary history and into the broader lifestyle strategies that enable Antarctic *Pseudomonas* lineages to persist in extreme polar ecosystems.

## MATERIALS AND METHODS

### Bacterial strains and growth conditions

*Pseudomonas* sp. AU10 was isolated by our research team from a freshwater sample collected at Uruguay Lake (Fildes Peninsula, King George Island, South Shetlands, Antarctica) (31). *Escherichia coli* ATCC 25922 (Genetic Stock Center) was used as a reference for biochemical tests; *E. coli* UT400 (K12, *entA403*, streptomycin-resistant), a mutant deficient in enterobactin production (36), served as a negative control for siderophore production assays; and *Herbaspirillum seropedicae* Z69, which produces both siderophores and polyhydroxyalkanoates (37), was employed as a positive control for testing the production of these compounds. UT400 and Z69 were kindly provided by M. Laviña (FGB, UdelaR) and S. Batista (BIOGEM, IIBCE), respectively. Bacterial cultures were routinely grown on LB agar (1% tryptone, 0.5% yeast extract, 1% NaCl, 1.5% agar, pH 7.4) or in LB broth and incubated at the appropriate temperature for each species (20 or 37 °C). Liquid cultures were shaken at 150 rpm.

### Genome sequencing, assembly, and annotation

Genomic DNA was extracted using the DNeasy Blood & Tissue Kit (Qiagen). Whole-genome sequencing was performed by Macrogen Inc. (Seoul, South Korea). Illumina TruSeq PCR-Free libraries were sequenced on a HiSeq 2000 platform, generating 101 bp paired-end reads, and PacBio SMRTbell libraries were sequenced on a Sequel IIe system to obtain HiFi long reads.. All subsequent analyses were performed in-house.

Illumina reads were quality-checked with FastQC (v0.11.6) and trimmed with Trim Galore (v0.6.10) using a minimum Phred score of 30, whereas PacBio HiFi reads were not further processed. Hybrid *de novo* assembly was performed using Unicycler (v0.4.8), with the minimum contig length set to 500 bp and all other parameters left at their default values (38). Within Unicycler, SPAdes assembled Illumina reads into contigs (optimal k-mer size of 89), Miniasm overlapped them with the long HiFi reads to construct the hybrid assembly, and Racon polished the assembly by aligning the long reads back to the draft in multiple rounds. For further refinement, Illumina reads were mapped back to the assembly using BWA (0.7.18), the alignments were processed with Samtools (1.21), and two iterations of Pilon (v.1.24) were performed to ensure high base accuracy (39). Assembly quality statistics were obtained with QUAST (v.5.2.0), closure was examined with Bandage (v.0.8.1), and completeness was assessed with BUSCO (v5.8.2) using pseudomonas_odb12 data set (40).

The complete genome of AU10 was annotated with the NCBI Prokaryotic Genome Annotation Pipeline (PGAP) during the GenBank submission process (accession JAIUXL020000001 for the chromosome and OQ302170 for the plasmid). An additional annotation was performed with RASTtk within the BV-BRC online server (https://www.bv-brc.org/) (41, 42). Predicted genes were functionally categorized in BV-BRC subsystems and in BRITE hierarchies, the latter obtained through the KEGG Automatic Annotation Server (KAAS), which also enabled the reconstruction of KEGG metabolic pathways (43, 44). KEGG orthologs were assigned using GHOSTZ and 40 reference genomes. Results obtained with BV-BRC subsystems, BRITE hierarchies, and KEGG pathways were interpreted using the MetaCyc database (https://metacyc.org/) to elucidate AU10’s metabolic networks (45). Prophages were predicted using PhiSpy within RASTtk.

### Taxonomic placement and comparative genomics

Different approaches were used to position AU10 taxonomically and identify the most similar genomes from public databases. First, an identification report was obtained from the Type Strain Genome Server (TYGS; https://tygs.dsmz.de/ ), based on the 16S rRNA gene and genome-scale analyses (46). Second, 1 Mb segments of AU10’s genome were BLAST-searched against the GenBank database, selecting genomes with the highest scores that appeared consistently across searches. Lastly, the “Similar Genome Finder” tool within BV-BRC, which uses the Mash/MinHash algorithm, was run against the BV-BRC bacterial genome database. Only genomes sharing at least 10% of their k-mers with the query were kept for further analysis. Genomic relatedness among the selected strains was assessed by calculating pairwise average nucleotide identity using BLAST (ANIb) with pyani (v0.2.12) (47).

### Phylogenomic analyses

The Codon Tree pipeline within the BV-BRC server was used to construct phylogenomic trees. The pipeline identified single-copy orthologous genes from distinct global protein families (PGFams) shared among the analyzed genomes. For each selected gene, the nucleotide and deduced amino acid sequences were retrieved and aligned with the Codon_align function of BioPython and MUSCLE, respectively. A maximum-likelihood phylogenomic tree was inferred using RAxML based on concatenated codon-aware alignments guided by protein sequences. The analysis was performed under a partitioned scheme, with a GTRCAT nucleotide substitution model applied separately to each codon position and a JTT protein substitution model applied to the protein partition (48). Node support was assessed using 100 bootstrap replicates. Trees were visualized in MEGA 11, and labels were edited in Inkscape (49). The list of orthologous genes used is available in the Zenodo repository (https://zenodo.org/records/17237991).

### Pangenomic analysis

A pangenome analysis was performed using Anvi’o (v.8) (50). Briefly, genomes were processed from nucleotide FASTA files to generate Anvi’o contig databases, in which genes were predicted and functionally annotated based on the NCBI COG20 database. Single-copy genes were then identified within each contig database by searching profile Hidden Markov models (HMMs) with HMMER. Finally, the contig databases were integrated, and a pangenome was constructed by identifying homologous genes with BLASTp (NCBI) and clustering them as COGs (Clusters of Orthologous Genes) by sequence similarity using the Markov Cluster Algorithm (MCL). Gene clusters were classified into core, accessory, and unique categories according to their distribution across genomes. The pangenome was manually divided into bins of gene clusters within the Anvi’o interface, based on presence–absence patterns, grouping genes with shared evolutionary relevance. Functional enrichment analysis was conducted to identify overrepresented COG functions within specific genome groups, as explained in Shaiber et al. (51). A function was considered enriched when the *q*-value (FDR-corrected *P*-value) was below 0.05.

To complement the cluster-based perspective provided by Anvi’o, proteomes of related strains were compared using the BV-BRC Proteome Comparison tool (42). Bidirectional BLASTp (≥80% identity, ≥80% coverage, E-value ≤ 1e-10) was applied to align protein sequences. Results were displayed in syntenic order relative to a reference genome, allowing visualization of proteins in genomic context rather than as isolated matches. The resulting comparison tables were exported and manually inspected to assess concordance across genome groups by identifying regions that were present or absent across them. To confirm these observations, the selected regions were examined with the BV-BRC Compare Region Viewer, using the “Selected genome group” option and the PLfam classification method, which enabled a clear visualization of gene conservation and variation in syntenic context across genomes.

### Phenotypic analyses

Standard biochemical tests were performed from fresh cultures using the following kits: API 20E (BioMérieux), HiCarbo Kit (KB009, HiMedia), HiAssorted Biochemical Test Kit (KB002, HiMedia), Hi24 Enterobacteriaceae Kit (KB016, HiMedia), and BD BBL Crystal Enteric/Nonfermenter ID Kit. All tests were performed following manufacturer’s instructions and were incubated at 28°C for at least 48 h before interpretation.

Lipolytic activity was assessed on tributyrin agar (TBA) plates (1% [vol/vol] tributyrin, 7.0 g/L peptone, 2.5 g/L soytone, 2.5 g/L NaCl, 18 g/L agar, pH 7) and on Tween 80 agar (T80A) plates (1% [vol/vol] Tween 80, 7.0 g/L peptone, 2.5 g/L soytone, 2.5 g/L NaCl, 0.1 g/L CaCl_2_, 18 g/L agar, pH 7) (52). Bacterial suspensions were inoculated on the plates and incubated at 28°C for 48 h. Esterase activity was detected on TBA plates by the appearance of clear hydrolysis halos after iodine staining, whereas lipase activity was indicated on T80A plates by the formation of an opalescent zone around colonies.

Cellulolytic activity was tested on carboxymethyl cellulose (CMC) agar plates (1.0 g/L CMC, 2.5 g/L NaNO_3_, 0.2 g/L MgSO_4_, 0.2 g/L NaCl, 0.1 g/L CaCl_2_.6H_2_O, 18 g/L agar, pH 7) (52). After 48 h of incubation at 28°C, plates were stained with 1% Congo red solution and destained with 1 M NaCl to reveal discoloration halos indicative of cellulase activity.

Siderophore production was assessed using Chrome Azurol S (CAS) agar medium and incubating the plates at 30°C, following the method of Schwyn & Neilands (53). Pyoverdine production was evaluated on King B agar (20 g/L proteose peptone No. 3, 10 g/L glycerol, 1.5 g/L K_2_HPO_4_, 1.5 g/L MgSO_4_·7H_2_O, 18 g/L agar) by observing fluorescence under UV light (54).

Polyhydroxyalkanoate (PHA) production was tested on M9 salts agar (55) supplemented with 4% glucose. After 48 h of incubation at 28°C, a 0.1% (wt/vol) Nile Red solution in acetone was added dropwise onto the surface of the grown colonies, and fluorescence was assessed under UV light. Orange-red fluorescence was interpreted as a positive result for PHA accumulation (56).

β-hemolysis was evaluated on 5% sheep blood agar plates, as described (57).

## RESULTS

### General features of the *Pseudomonas* sp. AU10 genome

The genome of AU10 was sequenced using Illumina and PacBio technologies, yielding 12,949,692 short reads (101 bp) and 90,592 long HiFi reads (938 Mb; N_50_: 11,449 bp). A hybrid *de novo* assembly was performed, with Illumina reads contributing high per-base accuracy and PacBio reads providing long-range continuity. The final assembly consisted of a circular chromosome (6,719,885 bp) and a circular plasmid (31,152 bp), thus achieving complete genome closure. Bandage visualization confirmed the absence of disconnected contigs. Sequencing coverage was approximately 194× for Illumina and 139× for PacBio. The chromosome GC content was 59.7%, consistent with the expected range for *Pseudomonas* species (58), and 54.9% for the plasmid. According to BUSCO analysis, the assembly was 99.2% complete, with 0.1% duplicated, 0.3% fragmented, and 0.5% missing genes, likely reflecting natural variation relative to the genus-level reference data set. Genome annotation identified 6,236 protein-coding sequences (CDSs), 71 tRNA genes, and 19 rRNA genes, corresponding to six complete rRNA operons plus an additional 5S gene. Functional annotation assigned putative functions to 76.7% of CDSs. Yet, only 30% were categorized into functional subsystems and 15% into KEGG pathways. These fractions provided insight into the strain’s metabolic and general capabilities.

### Mobilome contribution to AU10’s Antarctic adaptation

The 31 kb natural plasmid from AU10 (named pAU10) showed no full-length homologs in a BLAST search. The closest match was a 78 kb plasmid from *Pseudomonas lurida* L228, isolated from plant material in Ireland, with 88% identity and 43% query coverage. This suggests that pAU10 is a previously unreported Antarctic plasmid. It carries 46 CDSs (Fig. S1), most of which are related to plasmid metabolism, including replication (*repJ* and *repB*), partitioning (*parA* and *parB*), remnants of a conjugative *tra* region, and addiction systems (*relE*/*relB* and *chpS*/*chpB*). It also carries an O-antigen acetylase gene and the *umuDC* operon, which codes for an error-prone DNA polymerase involved in mutagenic repair during an SOS response triggered, for instance, by UV damage.

Additionally, six prophages were identified in the chromosome, ranging in size from 11 to 33 kb. Notably, one of them encodes an O-antigen acetylase, as does pAU10.

### A synopsis of AU10’s metabolic potential

**Fig 1:**
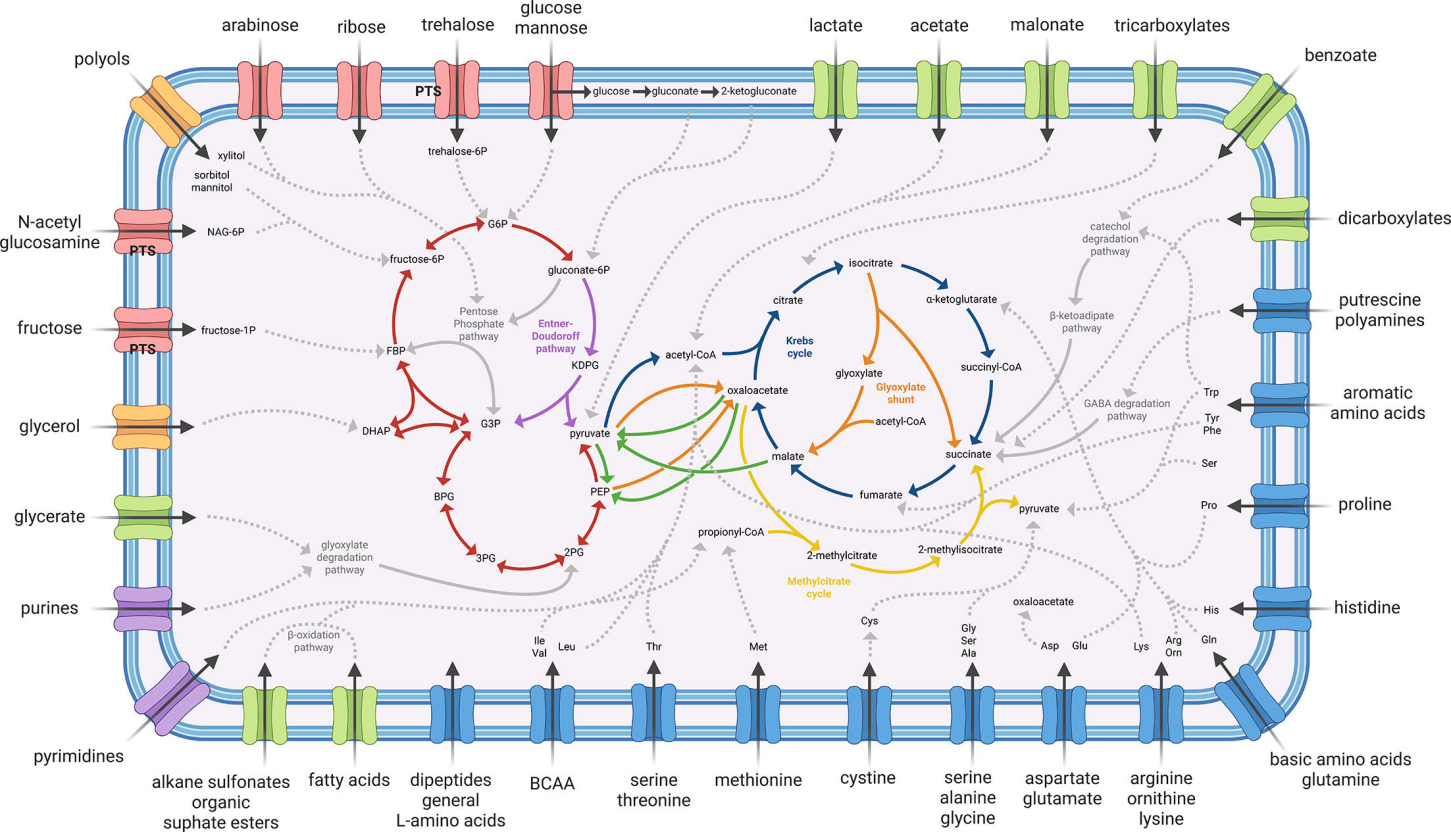
Metabolic model for *Pseudomonas* sp. AU10 carbon metabolism based on genomic and phenotypic information. A schematic cell is depicted with both membranes defining the cytosolic and periplasmic subcellular spaces. Transport systems are represented schematically, with different colors according to their specificity: sugars and derivatives in red; polyols in orange; organic acids in green; amino acids and derivatives in blue; and nucleotide bases in purple. Phosphotransferase transport systems are identified as PTS. Central carbon pathways are represented as solid-colored arrows: gluconeogenic and glycolytic reactions in red; Entner–Doudoroff pathway in purple; decarboxylation of pyruvate and the Krebs cycle in blue; the methylcitrate cycle in yellow; anaplerotic nodes including the glyoxylate shunt in orange; and cataplerotic/gluconeogenic interconnecting nodes in green. Deduced catabolic pathways for each carbon source are represented with dashed gray arrows showing the point of central carbon pathways at which they end. Solid gray lines represent pathways named in the figure. Abbreviations: 2PG, 2-phosphoglycerate; 3PG, 3-phosphoglycerate; BCAA, branched-chain amino acids; BPG, 2,3-bisphosphoglycerate; DHAP, dihydroxyacetone phosphate; FBP, fructose-1,6-bisphosphate; G3P, glyceraldehyde 3-phosphate; G6P, glucose-6-phosphate; KDPG, 2-dehydro-3-deoxy-6-phospho-gluconate; PEP, 2-phosphoenolpyruvate.

We reconstructed the metabolic pathways and transport systems of AU10 from its assembled genome to assess its metabolic potential. Fig. 1 illustrates the expected range of assimilable carbon and energy sources, along with the central carbon pathways through which they would be channeled. This metabolic model was partially supported by 64 standardized biochemical assays (Table S1), which confirmed AU10’s broad heterotrophic capacity.

Results presented in Fig. 1 suggest that AU10 is biased toward the catabolism of amino acids and organic acids over sugars and their derivatives. Most of its transporters are dedicated to small mono-, di-, and tricarboxylic acids, amino acids, fatty acids, and even detergents such as alkane sulfonates and alkyl/aryl sulfate esters (Fig. 1). In contrast, only a handful of ABC transporters and three phosphotransferase systems (PTS) were identified for sugars and related derivatives. This pattern matches what is observed in other *Pseudomonas* species, where catabolite repression favors the assimilation of organic acids and amino acids over sugars (59).

For glucose utilization, AU10 presents both the phosphorylative and oxidative pathways typical of *Pseudomonas*, which converge at 6-phosphogluconate (60). This intermediate can be further metabolized through either the Entner–Doudoroff or the pentose phosphate pathways (Fig. 1). As expected for the genus (60), the classical Embden–Meyerhof–Parnas glycolytic pathway is incomplete in AU10 due to the lack of 6-phosphofructokinase. The strain showed acid production from certain sugars (Table S1); however, since members of this genus are considered sugar non-fermentative (1), this phenotype is likely due to the promiscuous activity of periplasmic membrane-bound glucose dehydrogenase, as previously described (61). Thus, AU10 may oxidize glucose and other sugars at the periplasm through the oxidative route, releasing aldonic acids and thereby contributing to local acidification of the medium (60).

The respiratory chain of AU10 would be strictly aerobic and branched, comprising five complete terminal oxidases: three cytochrome c oxidases (types *aa3*, *cbb3*-1, and *cbb3*-2) and two ubiquinol oxidases (*bo* and *bd* types). These enzymes differ in oxygen affinity, redox potential, energy coupling efficiency, and tolerance to environmental stressors (62), suggesting that AU10 could grow under a wide range of oxygen concentrations. No genes involved in nitrate respiration or denitrification were detected, and no nitrate or nitrite reductase activity was observed (Table S1). Interestingly, AU10 has the *arcDCB* operon and exhibited arginine deiminase activity, confirming its ability to ferment arginine. In other *Pseudomonas* species, this pathway supports survival or even growth under transient anoxic conditions (63, 64). In contrast, pyruvate fermentation to lactate and acetate, a pathway present in several *Pseudomonas* strains (15, 65), appears to be incomplete in AU10 due to the absence of the acetate kinase gene.

Metabolic versatility in AU10 is underscored by the presence of several anaplerotic and cataplerotic/gluconeogenic nodes known to interconnect central carbon metabolism in bacteria (66). We identified three cataplerotic enzymes, which enable the use of di- and tricarboxylic acids and certain amino acids as energy or gluconeogenic sources: phosphoenolpyruvate (PEP) carboxykinase, NADP^+^-dependent malic enzyme, and oxaloacetate decarboxylase (green arrows in Fig. 1). The anaplerotic nodes identified in AU10, which replenish the Krebs cycle with intermediates, include a PEP carboxylase, a pyruvate carboxylase, and the complete glyoxylate shunt (orange arrows in Fig. 1). The shunt would enable this strain to meet its carbon requirements when growing on substrates that enter central pathways as acetyl-CoA, such as fatty acids. Additionally, AU10 has the full methylcitrate cycle (yellow arrows in Fig. 1), which converts the toxic intermediate propionyl-CoA into succinate and pyruvate (67). Altogether, these interconnecting nodes would allow AU10 to fine-tune its metabolism under different carbon regimes.

AU10’s reliance on amino and organic acids matches its hydrolytic enzyme repertoire. The strain was originally recognized for its proteolytic activity at low temperatures, and one of its secreted enzymes was biochemically characterized as a cold-active alkaline serine-metalloprotease (31, 33). Here, we identified more than 50 encoded proteases, along with several lipases, esterases, a chitinase, and a chitin-binding protein. On the bench, lipase/esterase activity was demonstrated on Tween 80 and tributyrin agar plates (Fig. S2). Conversely, we found no genes encoding enzymes for the degradation of disaccharides or complex plant polysaccharides, such as β-galactosidases, invertases, polygalacturonases, xylanases, arabinofuranosidases, endoglucanases, or cellobiohydrolases. These findings are consistent with the negative results on carboxymethyl cellulose plates and in several biochemical tests (Table S1). Thus, AU10 appears to lack the enzymatic machinery required to degrade lactose, sucrose, or major plant-derived polysaccharides, such as pectins, hemicellulose, and cellulose, consistent with a non–plant-associated lifestyle.

Concerning storage polymer metabolism, genome analysis revealed that AU10 harbors complete gene sets for the synthesis and degradation of glycogen, cyanophycin, polyphosphate, and polyhydroxyalkanoates (PHA). Its PHA gene cluster matches Class II systems typical of the genus *Pseudomonas*, which mediate the synthesis of medium chain-length PHAs (68). However, PHA production was not detected under the tested conditions, as assessed by Nile Red staining.

### AU10 belongs to a long-established Antarctic lineage

To clarify the taxonomic placement of AU10 within the genus *Pseudomonas*, we first submitted its genome sequence to the TYGS platform (46). The resulting report indicated that AU10 would be a novel species, as no type strain showed digital DNA–DNA hybridization (dDDH) values above the 70% threshold used for species delineation (69). The similarity matrix derived from dDDH values and the GBDP (Genome BLAST Distance Phylogeny) tree indicated that the closest described species were *Pseudomonas antarctica*, *Pseudomonas fluorescens*, *Pseudomonas salomonii*, and *Pseudomonas edaphica*. To gain further insight, we constructed a phylogenomic tree including AU10 and 40 type strains of validated *Pseudomonas* species, selected based on previously published phylogenies (70), and including all publicly available type-strain genomes of Antarctic *Pseudomonas* species (Fig. S3). The resulting tree confirmed AU10’s closest relationship with *P. antarctica*, followed by the three species mentioned above. The tree topology also revealed that Antarctic species are distributed polyphyletically across the genus, interspersed among species from other geographic regions.

To explore whether AU10 belongs to a coherent Antarctic lineage, we searched public databases for closely related genomes using both BLASTn and the “Similar Genome Finder” tool from BV-BRC. At the time of the analysis, both databases contained around 12,000 *Pseudomonas* genomes not belonging to the human pathogen *Pseudomonas aeruginosa*. Query genomes in both searches included AU10, isolates previously assigned to *P. antarctica* (BS2772^T^, S101, 03-1.1-1.2, and PAMC 27494), and the type strain of *P. fluorescens* (ATCC 13525^T^). This approach enabled us to identify 40 non-redundant genomes closely related to AU10, all of which fell within the clade outlined by a dashed rectangle in Fig. S3.

**Fig 2:**
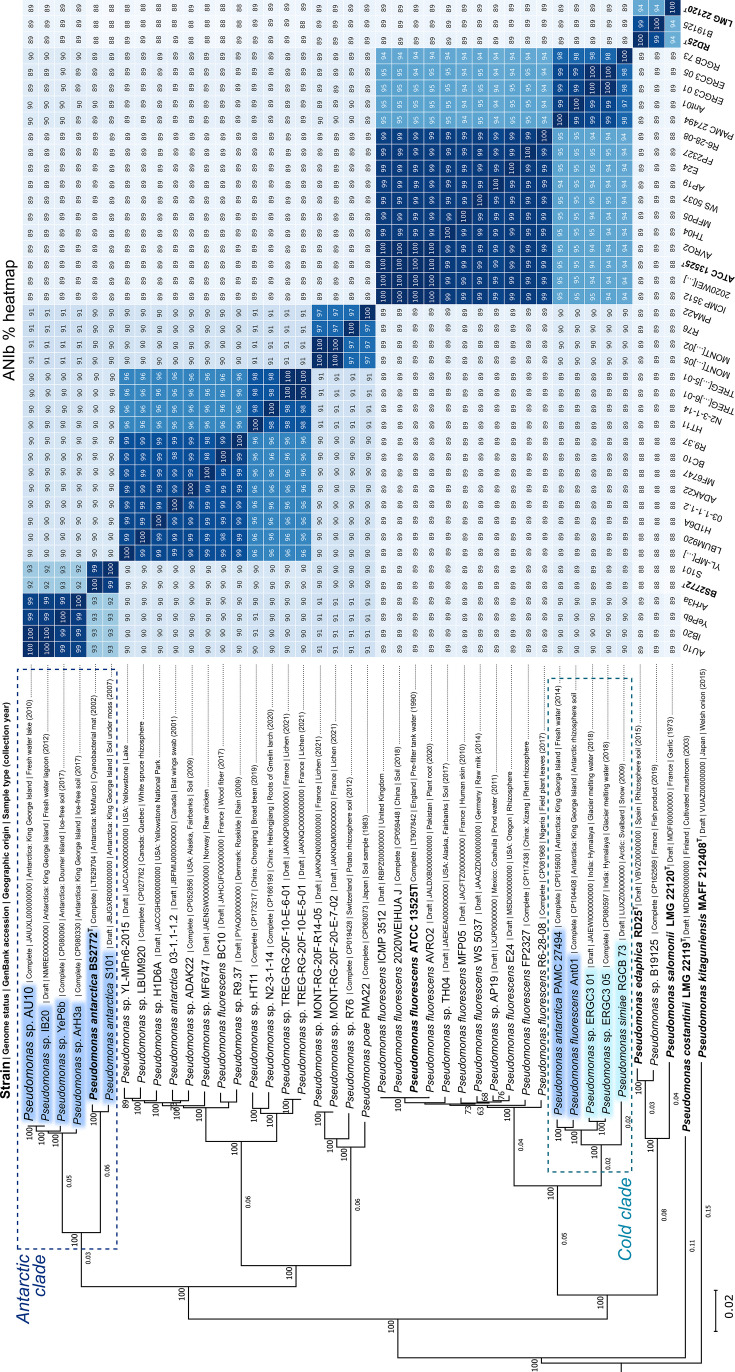
Phylogenomic tree and pairwise average nucleotide identity (ANIb) heatmap of *Pseudomonas* sp. AU10 and closely related genomes. The maximum-likelihood tree, showing branch lengths in substitutions per site, was inferred from 959 single-copy orthologous genes. Bootstrap support values (100 replicates) are shown at the nodes. *P. kitaguniensis* and *P. constantinii* type strains were included as outgroups. Type strains are shown in bold; strains from Antarctica are shaded in blue; and strains from non-Antarctic freezing environments are shaded in light blue. Metadata for each strain is indicated. Dashed rectangles highlight two separate monophyletic clades, the “Antarctic clade” and the “cold clade,” each comprising isolates from extremely cold environments. The adjacent heatmap shows pairwise ANIb values (%), with darker shading indicating higher nucleotide identity. Each cell represents a pairwise comparison between the named genomes on rows and columns.

To further resolve AU10’s relationship to these 40 similar strains, we performed a second phylogenomic analysis and calculated pairwise average nucleotide identity (ANIb) values. *Pseudomonas kitaguniensis* and *Pseudomonas constantinii* type strains were included as outgroups because they represent the closest species external to the focal group, based on their placement in our initial tree (Fig. S3) and published phylogenies (70, 71). Results are shown in Fig. 2, together with relevant metadata from the 43 strains included in the analysis. Nearly half of these lacked formal species-level assignments, and only seven originated from Antarctica. The tree revealed that AU10 and five of the seven Antarctic strains (IB20, YeP6b, ArH3a, BS2772^T^, and S101) formed a monophyletic clade supported by 100% bootstrap values. This group, hereafter termed “AU10’s Antarctic clade” or simply the “Antarctic clade,” was the only lineage in the tree that combined phylogenetic cohesion with a clear geographic origin and is outlined by a dashed rectangle in Fig. 2. In contrast, all other clades showed geographically mixed compositions (see figure metadata). The Antarctic clade was also characterized by relatively long branches in the tree, suggesting a prolonged period of divergence from its closest relatives. One observation that drew our attention was the absence of South American genomes among AU10’s closest relatives, despite the geographic proximity of South America to the Antarctic Peninsula. At the time of analysis, the BV-BRC database contained 928 *Pseudomonas* genomes from South America (excluding *P. aeruginosa*) and only 125 from Antarctica. In contrast, closely related strains were retrieved from comparatively under-sampled regions such as Nigeria and Pakistan. Taken together, these observations are consistent with the putative endemism of the Antarctic clade.

Regarding the ecology of the clade, almost all members were recovered from freshwater or soil samples, suggesting an adaptation to terrestrial Antarctic habitats. Only the origin of IB20 is somewhat ambiguous: while its GenBank entry (2017) classifies it as a freshwater isolate, the published report (2018) describes it as marine (72) in the context of a survey that included both freshwater and marine environments. The first strain described was *P. antarctica* BS2772^T^, collected in 2002 from a cyanobacterial mat in the McMurdo Dry Valleys. The remaining members were isolated more recently and further west from islands surrounding the Antarctic Peninsula (72, 73).

Within the Antarctic clade, ANIb values displayed as a heatmap in Fig. 2 revealed two distinct species-level subgroups, based on the 95% identity threshold commonly applied for species delineation (69). One corresponded to *P. antarctica*, represented in our data set by strains BS2772^T^ and S101. The other species included AU10, IB20, YeP6b, and ArH3a, which form a well-defined cluster in both the phylogenetic tree and the ANIb matrix. Very recently, the name *Pseudomonas gelidaquae* was proposed for this species, with IB20 designated as the type strain (71). Although formal validation is still pending, we will refer to this lineage hereafter as *P. gelidaquae* or “AU10’s species.”

The two remaining Antarctic strains in our data set (PAMC 27494 and Ant01) did not cluster within AU10’s Antarctic clade. To our surprise, they grouped with three strains isolated from samples collected in remote, permanently cold environments, including glacier meltwater in the Himalayas and Arctic snow from Svalbard (74, 75). These geographically distant yet ecologically similar strains clustered into a distinct monophyletic group, hereafter termed the “cold clade,” highlighted by a dashed rectangle in Fig. 2. Although this clade was phylogenetically close to *P. fluorescens* species, ANIb values among members of both groups ranged from 94% to 95% (Fig. 2), placing the cold clade at the boundary of species delimitation. These results also indicated a species misassignment for PAMC 27494, which would not belong to *P. antarctica*. The remaining strains in the data set (not belonging to the Antarctic or cold clades) originated from warmer regions, except for a few from seasonally cold areas that experience warm summers, such as Fairbanks, Alaska, and therefore cannot be considered permanently cold environments.

### Pangenomic insights into the evolution of AU10’s lineage

**Fig 3:**
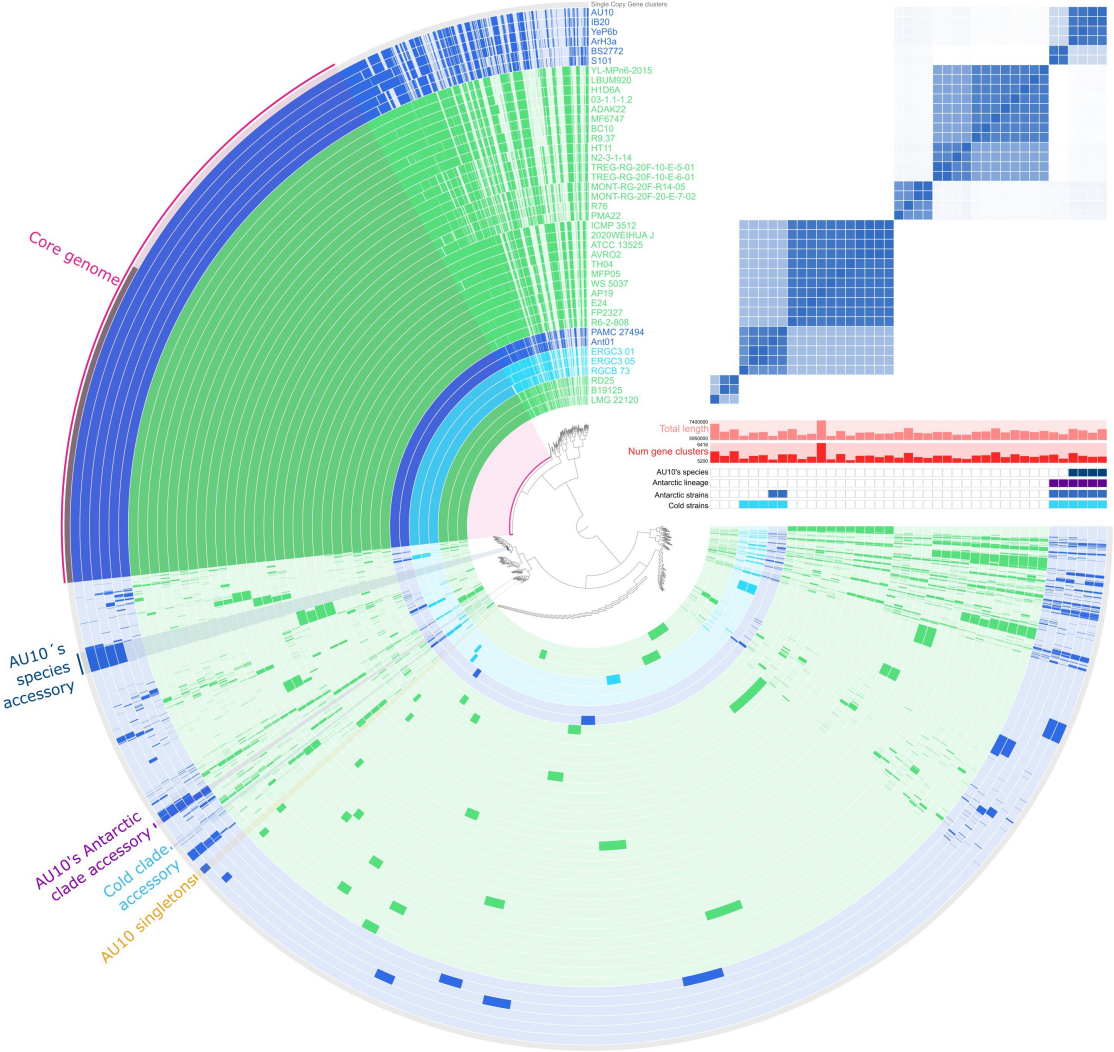
Pangenome of *Pseudomonas* sp. AU10 and 40 related strains. The central dendrogram organizes gene clusters (GCs) by their presence/absence patterns across genomes. Concentric rings indicate the presence (colored) or absence (pale shade) of each GC in a given genome. Bins of GCs, defined by their distribution patterns, are shown as shaded radial bands: core genome (magenta), Antarctic clade–specific (purple), cold clade–specific (cyan), accessory to AU10’s species (blue), or AU10 singletons (orange). Genome order follows the phylogenomic tree in Fig. 2. The top-right panel shows an ANIb heatmap (mirror image of Fig. 2) illustrating pairwise genomic similarity.

Based on the results presented above, we asked whether the Antarctic and cold clades evolved specific adaptations to freezing environments. To investigate these putative lineage-specific signatures of adaptation, we performed a pangenomic analysis with Anvi’o, built on clusters of orthologous genes (COGs) from the genomes of AU10 and the 40 closely related strains identified earlier (Fig. 3; Table Z3 at Zenodo: https://zenodo.org/records/17237991). The resulting pangenome revealed that the core genome, shared by all analyzed strains, accounted for only 25% of the total COGs (3,901 out of 15,625). In contrast, 39% corresponded to accessory clusters shared by at least two strains (6,072 clusters), while 36% were singletons (5,652 clusters) present in only one genome (Fig. 3). Within this framework, we identified blocks of accessory gene clusters specific to AU10’s Antarctic clade, the cold clade, AU10’s species, and AU10 singletons. These blocks were binned using Anvi’o and are indicated with radial color shading in Fig. 3, while their constituent gene clusters are listed in Table Z3 in the Zenodo repository. To interpret this variability in an evolutionary context, gene cluster presence–absence patterns were examined in light of the phylogenetic topology shown in Fig. 2.

**Fig 4:**
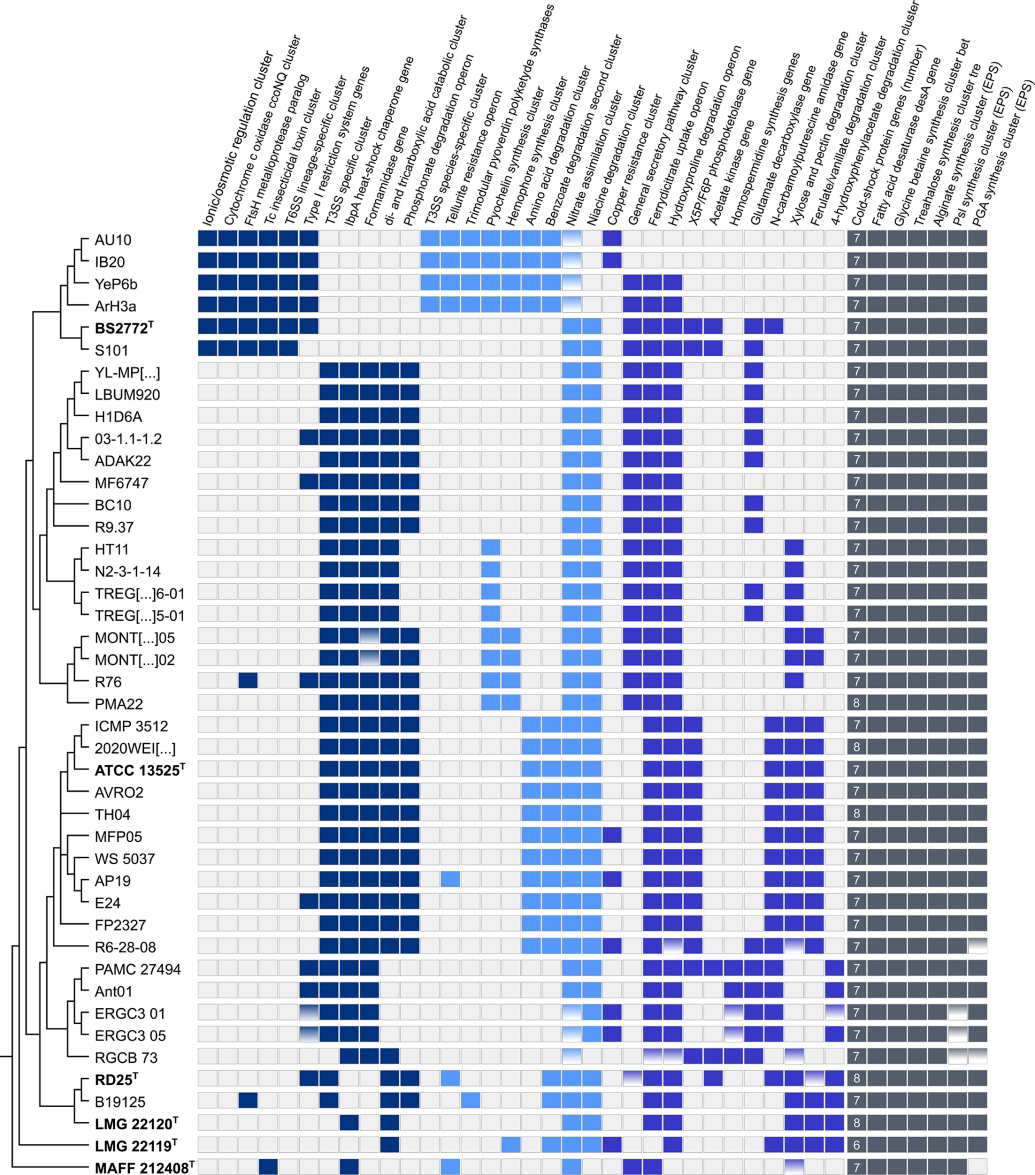
Distribution of selected genomic features across close relatives of AU10. The phylogenomic topology (from Fig. 2) is shown on the left. Colored squares indicate feature presence, with color patterns reflecting features distinctive of particular groups: dark blue for AU10’s Antarctic clade, light blue for AU10’s species, purple for features without clade-specific distribution, and dark gray for genes commonly associated with cold adaptation. The features shown were identified through pangenomic analyses, and their genomic integrity was visually and syntenically verified using the *Compare Region Viewer* tool from the BV-BRC server. Gradient-filled squares indicate gene decay, including premature stop codons or partial remnants.

To support the assignment of bins and gain further insight into their biological relevance, we combined two complementary approaches. Functional enrichment analyses in Anvi’o identified functions significantly overrepresented in particular groups of strains (Tables Z4–Z7 at Zenodo: https://zenodo.org/records/17237991). To reduce potential biases due to unequal group sizes, only functions broadly represented within each group were retained for interpretation. In turn, alignments generated with the “Proteome Comparison” and the “Compare Region Viewer” tools in BV-BRC displayed gene gains and losses in syntenic order relative to a reference genome (AU10, ATCC 135225^T^, and PAMC 27494; Tables Z8–Z10 at Zenodo). This provided genomic context to determine whether they corresponded to genomic islands, mobile elements, or functionally coherent clusters restricted to specific lineages. The most relevant findings obtained from these complementary strategies are summarized in Fig. 4 and further developed below in the form of key questions that guide the presentation of our results.

### Are there genomic hallmarks of freeze adaptation?

When comparing the 11 strains from the Antarctic and cold clades collectively with the remaining 30 strains not associated with permanently cold environments, no accessory gene clusters or functions were found to be exclusively shared or absent. Functional enrichment did identify significant COG functions, but only as partial enrichments rather than absolute 11-versus-30 exclusivity. Syntenic proteome comparisons likewise did not reveal genomic regions exclusive to these groups. Together, these results indicate that cold adaptation does not rely on a single convergent set of accessory genes.

On the other hand, three gene clusters were absent in both the Antarctic and cold clades, although not exclusively. These included a genomic island encoding a TRAP-type C4-dicarboxylate transport system and a citrate lyase, involved in dicarboxylate uptake and metabolism; the *phn* operon responsible for phosphonate degradation; and a gene cluster involved in xylose and pectin degradation (Fig. 4).

We also examined well-described genetic determinants associated with bacterial survival at low temperatures, including cold-shock protein genes (*csp*), the fatty acid Δ9-desaturase gene (*desA*), and genes involved in the synthesis of cryoprotectant molecules, such as compatible solutes and exopolysaccharides (EPS) (15, 17, 18, 76). We found that all analyzed genomes encoded multiple *csp* paralogs, and copy number did not correlate with isolation from permanently cold environments (Fig. 4). Likewise, the *desA* gene, important for preserving membrane homeoviscosity under cold conditions (76), was present in all strains. All genomes also carried genes for cryoprotectant compatible solutes (15, 76), including the *betIBA* operon for glycine betaine synthesis from choline and *treYZXS* for trehalose production from maltose and glycogen (Fig. 4), whereas the *otsAB* operon for *de novo* trehalose synthesis was absent in all genomes. Finally, EPS gene clusters for alginate, Psl, and poly-β−1,6-N-acetyl-D-glucosamine (PGA) synthesis were present in almost all strains (Fig. 4). In sum, these canonical cold-associated genes were widespread and did not distinguish strains from permanently cold environments from closely related strains inhabiting warmer habitats.

### What distinguishes AU10’s Antarctic clade?

The Antarctic clade was distinguished from the remaining 35 related strains by a set of accessory gene clusters uniquely shared among its six genomes (Fig. 3 and 4). Notably, it carries a genomic island encoding multiple proteins involved in ionic and osmotic regulation, including Na^+^/H^+^ antiporters (NhaA and NhaP), a H^+^/Cl⁻ antiporter (ClcA), a K^+^ uptake protein (TrkH), and a choline–glycine betaine transporter (BetT). These genes represent additional copies of canonical variants that are typically dispersed across the chromosome, rather than organized in clusters. It also carries a distinctive *ccoNQ* operon, which encodes two subunits of a high-affinity Cbb3-type cytochrome c oxidase. This operon adds to the two canonical *ccoNOQP* versions (*cbb3*-1 and *cbb3*-2), which are conserved across all 41 strains and function as branched terminal oxidases in the respiratory chain. The Antarctic clade also differs in genes potentially associated with the heat-shock response: it lacks *ibpA*, which encodes a small ATP-independent heat-shock chaperone that prevents irreversible protein aggregation during heat stress, and harbors an additional copy of the *ftsH* gene (Fig. 4). The canonical *ftsH* encodes a membrane-bound, ATP-dependent protease that targets the heat-shock sigma factor RpoH for degradation under non-stress conditions (77).

The remaining features associated with AU10’s Antarctic clade correspond to virulence-related traits typically linked to pathogenicity. In particular, the clade has acquired a gene cluster encoding a type VI secretion system (T6SS), which mediates the injection of effector proteins into target cells (78), as well as a genomic island encoding insecticidal toxins of the Toxin Complex (Tc) type. The Tc locus identified (~18 kb) contains five genes encoding large proteins (800 to 1,600 amino acids) and shows the same organization as type V and type VI Tc clusters known to confer insect toxicity in other *Pseudomonas* species (79). These proteins assemble into a giant pore-forming complex that delivers a lethal cytotoxic domain into target cells (80). Of note, both species within the Antarctic clade harbor distinct versions of the Tc cluster. A BLASTn comparison between the loci of AU10 and *P. antarctica* BS2772^T^ showed only 71% query coverage and 83% nucleotide identity, whereas the AU10 cluster displayed much higher similarity to 11 distantly related *Pseudomonas simiae* strains, with 100% coverage and 91% identity in all cases (see phylogenetic distance in Fig. S3). In both Antarctic species, the Tc cluster is inserted at the same chromosomal site, with no mobile elements flanking it.

### Which accessory genes define AU10’s Antarctic species?

The four strains of *P. gelidaquae* (AU10, IB20, ArH3a, and YeP6b) shared a distinct set of accessory genes, as summarized in Fig. 4. In addition to the distinct Tc toxin cluster, all strains encode a complete type III secretion system (T3SS), known to inject effector proteins into eukaryotic host cells (81). Here, we found that most other closely related strains encode an alternative T3SS variant (Fig. 4), except for *P. antarctica,* which lacks both T3SS variants.

Additional acquisitions are related to iron uptake. In this regard, AU10 is a siderophore-producing bacterium, as evidenced by the formation of a clearance halo around stabs on CAS agar plates (Fig. S2A). More specifically, it secreted pyoverdine when grown on King B agar (Fig. S2B), a yellow-green siderophore that fluoresces under UV light. Pyoverdine production is a hallmark of fluorescent *Pseudomonas* (1), but its structure varies among species due to differences in the peptide backbone linked to the chromophore. These peptides are synthesized by different combinations of non-ribosomal peptide synthetases (NRPS) encoded in pyoverdine biosynthesis clusters (82). Here, we found that all strains of *P. gelidacquae* harbor a distinct set of three NRPS genes, while almost all remaining strains harbor only two (Fig. 4), consistent with the production of a structurally distinct pyoverdine. Moreover, the four genomes of *P. gelidaquae* gained two further gene clusters encoding all the necessary components for the synthesis, excretion, and uptake of the siderophore pyochelin and the hemophore HasA. While pyochelin mediates Fe^3+^ uptake, HasA scavenges heme from extracellular hemoproteins, and both are considered virulence factors in *P. aeruginosa* (83).

AU10’s species also acquired two catabolic operons involved in amino acid metabolism and benzoate degradation, while losing the complete cluster for nicotinic acid catabolism. The amino acid operon includes additional copies of proline dehydrogenase (PutB), threonine aldolase, and cysteine desulfurase genes, supplementing those present in the core genome. The benzoate degradation operon likewise represents a complete copy, in contrast to the canonical cluster, which lacks both the gene encoding the specific outer membrane porin and the terminal enzyme of the pathway, benzoate diol dehydrogenase (BenD).

An additional species-specific trait is the tellurite resistance operon (*terZABCDE*), which could expand its resistome against this widespread metalloid. Additionally, all four genomes lack the genes for assimilative nitrate and nitrite reductases and carry nonsense mutations in the nitrate-specific transporter genes (Fig. 4). As already mentioned, AU10 showed no detectable nitrate or nitrite reductase activity (Table S1).

### Does AU10’s species show variation at the strain level?

Some genomic signatures were not shared among all *P. gelidaquae* strains (AU10, IB20, ArH3a, and YeP6b), providing evidence of intraspecific variability. Notably, AU10 and IB20 lost a large genomic island present in closely related strains, which contains genes for the general secretory pathway (type II secretion system, T2SS) and the *fecBCDE* operon, encoding an ABC-type transporter responsible for ferric citrate uptake as a siderophore (Fig. 4). Despite lacking the *fec* operon, both AU10 and IB20 retained the *fecA* gene, which encodes the outer membrane porin that binds ferric citrate. In contrast, both strains acquired a copper resistance gene cluster previously linked to AU10’s copper resistance phenotype (35).

Other major contributors to intraspecific variability were the genomic islands carrying O-antigen biosynthetic loci, which determine the outermost and most variable portion of the outer membrane lipopolysaccharides (LPS). All four *P. gelidaquae* genomes contained two O-antigen clusters, but their composition and distribution varied across strains. One of them corresponded to an OSA cluster, a common variant among *Pseudomonas* species and determinant of serotype in *P. aeruginosa* (84). The same OSA locus was present in AU10, IB20, and YeP6b, while ArH3a carries a different variant at the same genomic location.

The second O-antigen cluster, present in AU10 and IB20 but absent from YeP6b and ArH3a, was rare among *Pseudomonas* genomes. BLASTn analysis of AU10’s entire cluster identified closest homologs in two related strains—*Pseudomonas* sp. PMA22 and *Pseudomonas* sp. R76 (86% identity, 63% query coverage; see relatedness in Fig. 2)—as well as in several distant *Pseudomonas protegens* strains (~80% identity, ~35% query coverage; see phylogenetic distance in Fig. S3). The best-characterized homolog cluster was found in *P. protegens* CHA0, an entomopathogenic model strain, where it was shown to be responsible for the production of long O-antigen polymers called OBC3 (85). Based on gene content and homology, we classified the AU10/IB20 locus as OBC3-like. In Fig. S4, we show the similarity blocks of AU10’s OBC3-like and OSA-like clusters compared to *P. protegens* CHA0 and *P. aeruginosa* PAO1.

Interestingly, all 43 closely related strains here analyzed harbored an OSA variant always located between the conserved chromosomal genes *ihfB* and *wbpM*. By contrast, only 12 strains carried an O-antigen island at the same chromosomal site as AU10’s OBC3-like locus, located between the CAAX amino-terminal protease gene and the aerotaxis sensor receptor gene, although the encoded clusters differed from OBC3. Moreover, neither locus was flanked by mobile genetic elements.

## DISCUSSION

This study aimed to elucidate the lifestyle and evolutionary history of *Pseudomonas* sp. AU10 by integrating genome-based approaches with targeted phenotypic assays. To this end, we sequenced the strain’s genome, reconstructed its metabolic potential, and performed phylogenomic and pangenomic analyses. After placing AU10 within the broader phylogenetic framework of the *Pseudomonas* genus, we narrowed our comparative genomic analysis to a phylogenetically focused data set comprising AU10 and its closest relatives with publicly available genomes. This strategy enabled the resolution of fine-scale evolutionary signals that would likely be less apparent at broader phylogenetic scales. Accordingly, our approach differs from genus-wide pangenomic studies (3, 30).

From the broader phylogenomic analysis (Fig. S3), AU10 was found to belong to one of several Antarctic lineages interspersed among species from other geographic regions. This polyphyletic pattern suggests repeated, independent colonization of Antarctica by members of this genus, rather than radiation from a single ancestral founder. Therefore, AU10’s Antarctic clade represents the outcome of one of several evolutionary incursions that overcame both the geographic isolation and the extreme environmental conditions of the Antarctic continent. A similar scenario has recently been described for *Polaromonas*, in which multiple lineages would have independently colonized polar regions and diversified (29). In addition, our pangenomic analysis of closely related strains showed that canonical genes encoding functions commonly associated with cold tolerance—such as cold-shock proteins, fatty acid desaturases, and pathways for compatible solute and exopolysaccharide synthesis—are not restricted to lineages from permanently cold environments, but are also present in strains isolated from temperate and even warm habitats. This agrees with previous studies indicating that many *Pseudomonas* lineages are intrinsically psychrotolerant and already harbor the genetic capacity required for growth at low temperatures (86). Indeed, fluorescent *Pseudomonas* are well known for their ability to grow at refrigeration temperatures and for their frequent occurrence as spoilers of chilled foods (87). Together, these observations suggest that intrinsic psychrotolerance likely facilitated multiple independent colonization events of Antarctic environments.

Within the phylogenetically focused data set, AU10’s Antarctic lineage emerged as a coherent monophyletic clade (Fig. 2) composed of six strains distributed into two species, *P. antarctica* and *P. gelidaquae*, the latter being a recently described species to which AU10 belongs (71). Notably, this was the only clade combining a clearly geographically restricted distribution with relatively long branches, together pointing to long-term Antarctic endemism. Moreover, because bacterial generation times are expected to be considerably longer in permanently cold environments (88), branch length comparisons within the tree may underestimate the actual temporal depth of divergence, further supporting a prolonged evolutionary history in Antarctica. In parallel, our analysis identified a second monophyletic clade composed of strains isolated from permanently cold environments across a broad geographic range, including the Arctic, Antarctica, and Himalayan glaciers. The wide geographic distribution and ecological coherence of this “cold clade” suggest an evolutionary trajectory shaped by long-range dispersal and environmental filtering under cold extremes. As such, it exemplifies the Baas–Becking principle—“everything is everywhere, but the environment selects”—and aligns with niche-based models of microbial biogeography (89–91). In contrast, AU10’s Antarctic clade appears to reflect a history shaped by either limited dispersal or strong environmental filtering outside Antarctica, resulting in its restricted distribution. Thus, our findings revealed two closely related *Pseudomonas* lineages adapted to freezing conditions but exhibiting sharply contrasting biogeographic patterns.

Despite being native to freezing environments, our pangenomic analysis found no evidence of a distinctive gene set shared between the two clades that could be considered as a common toolkit for adaptation to permanently frozen habitats. Instead, their genomic repertoires point to independent evolutionary trajectories shaped by distinct combinations of gene acquisition and loss. These findings underscore the importance of interpreting bacterial adaptation to polar environments through the lens of lineage-specific evolutionary histories, an idea previously proposed (92, 93), but here supported by evidence from two very closely related clades.

Focusing on AU10’s Antarctic clade, we identified several genomic features that differentiate it from its closest relatives and may have contributed to its evolutionary success in Antarctica, including those related to ionic and osmotic regulation, aerobic respiratory metabolism, and heat-shock response. Of note, we found a distinctive genomic island encoding redundant systems for potassium, sodium, chloride, and glycine betaine homeostasis. Such redundancy is likely to enhance fine-scale control of osmotic and ionic balance, a strategy repeatedly highlighted as central to cold adaptation (15, 27, 29). In line with this, Raiger et al. (2015) reported that *Pseudomonas extremaustralis* carries a genomic island encoding redundant potassium homeostasis systems and emphasized their role in coping with osmotic stress in Antarctic environments (15). In parallel, the acquisition of an incomplete *ccoNQ* operon lacking the *OP* genes may expand the functional versatility of the respiratory chain in the Antarctic clade. In *P. aeruginosa* PAO1, similar incomplete operons contribute to the formation of alternative cbb3-type cytochrome c oxidase isoforms with distinct physiological roles by pairing orphan NQ subunits with canonical OP subunits. For instance, one such isoform has been shown to contribute to cyanide resistance, biofilm development, and virulence in a nematode infection model (94, 95). Regarding heat-shock-related genes, the Antarctic clade is distinguished from its closest relatives by the absence of the *ibpA* gene, which encodes a small heat-shock chaperone. In *P. putida*, deletion of *ibpA* causes severe growth defects at high temperatures, underscoring its key role in heat tolerance (96). Under permanently cold conditions, however, this chaperone may have become dispensable.

Below the clade level, we identified genomic features that are either characteristic of AU10’s species or variable within it, and which may be relevant for shaping adaptation to the Antarctic environment. Several acquisitions point to metal homeostasis as a major selective pressure. For instance, *P. gelidaquae* acquired multiple gene clusters related to iron uptake, including those involved in the synthesis of the pyochelin siderophore and the HasA hemophore, as well as a replacement of the NRPS portion of its pyoverdine biosynthetic cluster, which would likely result in the production of a structurally distinct siderophore variant. Butaite et al. (97) showed that strong competition for iron among soil and freshwater *Pseudomonas* strains promotes cheating behaviors through heterologous pyoverdine exploitation, thereby favoring structural diversification as a mechanism to avoid siderophore piracy (98). Thus, by producing a structural variant, *P. gelidaquae* might be better able to compete with other community members, including its sister species, *P. antarctica*. Together, these features suggest that iron availability represents an important ecological constraint in the niche occupied by AU10’s species. On the other hand, all *P. gelidaquae* strains acquired a tellurite resistance operon (*terZABCDE*), whereas only a few carry a copper resistance genomic island. In line with this, we previously showed that AU10 displays resistance to Cr(VI), Cu(II), Mn(II), Fe(II), and As(V), describing these loci as part of a broader set of genomic resistance determinants (35). However, AU10 exhibited a tellurite-sensitive phenotype under the conditions tested, suggesting the operon might be inactive or functionally repurposed. Notably, homologous operons have also been associated with resistance to phagocytosis, bacteriophage infection, and oxidative stress in *Pseudomonas citronellolis* (99).

Genomic plasticity in AU10 reflects additional selective pressures operating within this species. The strain carries a hitherto unreported natural plasmid (pAU10), whose most striking feature is the presence of the *umuDC* operon, encoding a DNA polymerase involved in mutagenic DNA repair during the SOS response. Plasmids harboring the *umuDC* operon have been directly associated with UV-resistant phenotypes (97), suggesting that pAU10 may contribute to coping with the intense ultraviolet radiation characteristic of Antarctic environments (100). In addition, O-antigen acetylase genes were detected in two mobile genetic elements of AU10 (pAU10 and a prophage), suggesting that recent selective pressures favor modification of the outer portion of lipopolysaccharides, the frontline of cellular exposure and defense. In *E. coli*, for example, O-antigen acetylation reduces outer membrane permeability, decreases susceptibility to bacteriophages, and increases resistance to lysozyme-mediated lysis (101). In this context, we found further intraspecific variability in O-antigen biosynthesis gene clusters among *P. gelidaquae* strains. All four strains carry one of two OSA-type clusters, and only AU10 and IB20 harbor a second O-antigen cluster corresponding to an OBC3-like locus, similar to one described in an entomopathogenic strain of *P. protegens* (85). Importantly, we found that each O-antigen locus is consistently inserted at the same chromosomal position and flanked by conserved genes, a pattern that extends across the remaining closely related strains analyzed. Similar modular exchanges of O-antigen islands have been reported previously in pathogenic *Pseudomonas* species and were attributed to recurrent replacement events mediated by horizontal transfer and homologous recombination (102, 103). The maintenance of O-antigenic diversity among conspecific strains suggests that balancing selection is at play, likely driven by fluctuating ecological pressures, such as phage recognition or predatory and immune evasion (103, 104). Our results show that comparable genome plasticity affecting O-antigen loci also shapes Antarctic environmental species.

Several of the clade- and species-specific features discussed above highlight the role of biotic interactions in shaping the evolutionary trajectories of these Antarctic lineages, particularly in the context of competition for limited resources and evasion of parasites or predators. However, a striking finding was that many of the key acquisitions distinctive of these groups correspond to genomic clusters encoding well-known virulence factors. For instance, the Antarctic clade acquired two genomic islands: one encoding insecticidal toxins of the Toxin Complex (Tc) type and the other encoding a type VI secretion system (T6SS). In turn, *P. gelidaquae* differs from *P. antarctica* by the presence of key virulence genes, including the above-mentioned pyochelin and hemophore (HasA) synthesis clusters, as well as a genomic island encoding a distinct type III secretion system (T3SS), which was recognized in the recent description of *P. gelidaquae* as a key feature of the species (71). Moreover, we found that both species harbor different versions of the Tc toxin cluster at the same chromosomal site, with the *P. gelidaquae* variant showing higher similarity to homologous clusters from distantly related *Pseudomonas* strains. This points to an ancestral acquisition followed by replacement through horizontal transfer and recombination. Taken together, the acquisition and divergence of virulence genes suggest that pathogenic traits have been under sustained selective pressure since the ancestral establishment of the clade in Antarctica and continued throughout its diversification process. We further propose that differences in virulence gene repertoires between *P. antarctica* and *P. gelidaquae* may have favored specialization toward different hosts, reflecting niche segregation within their shared Antarctic environment and potentially contributing to an ecological speciation process.

If the Antarctic clade indeed exhibits a pathogenic lifestyle, a key question concerns the identity of its potential hosts. Although direct experimental evidence is currently lacking, multiple lines of genomic, metabolic, and environmental evidence converge on a plausible hypothesis. At the genomic level, a central clue is provided by the presence of insecticidal toxins, whose name directly implicates arthropods and which have been extensively characterized in entomopathogenic bacteria (79, 105). In line with this, type III and type VI secretion systems—such as those present in AU10’s Antarctic clade or species—have been shown to play key roles in insect infections mediated by *Pseudomonas* and *Photorhabdus* strains (78, 106). Although this points to insects as potential hosts, the Antarctic environment is nearly devoid of them, with only two native species reported (107). Because AU10 and other clade strains were isolated from freshwater environments, we instead propose a potential pathogenic interaction with freshwater arthropods inhabiting Antarctic freshwater systems (108). Supporting this view, divers from the Artigas Antarctic Scientific Base have informally reported remarkably dense swarms of crustaceans in Uruguay Lake, the isolation source of AU10 (31). Moreover, fluorescent *Pseudomonas* populations in this lake have been shown to be particularly abundant, reaching approximately 10⁴–10⁵ CFU mL⁻¹ over a five-year survey (109). We consider this spatial co-occurrence to provide ecological support for the proposed interaction. For AU10, the associated lifestyle is further supported by its hydrolytic profile, which is oriented toward the degradation of proteins, lipids, and chitin, along with a bias toward amino acid and organic acid uptake, as inferred from its metabolic reconstruction (Fig. 1). This combination suggests the capacity to exploit the major components of zooplankton biomass through extracellular hydrolysis and subsequent uptake of the released products, including N-acetylglucosamine. In addition, the potential to synthesize multiple storage polymers (glycogen, cyanophycin, polyphosphate, and PHA) may allow AU10 to persist as dormant planktonic propagules between infection events, while the versatility of its respiratory chain and the capacity for arginine fermentation could enable survival across distinct redox conditions within the water column. More broadly, a life cycle tightly coupled to local arthropods could help explain the restricted geographic distribution of the Antarctic clade. In contrast, members of the cold clade—lacking these virulence determinants—may rely on a more flexible, free-living lifestyle, facilitating the colonization of cold environments worldwide. These contrasting ecological strategies could therefore underlie the distinct biogeographic patterns observed. Future work using appropriate infection models will be essential to test these hypotheses.

To conclude, this work illustrates how integrating metabolic reconstruction, phylogenomics, and pangenomics within a restricted evolutionary framework can illuminate the lifestyles of environmental bacteria that are otherwise difficult to infer. While genome-based metabolic reconstructions delineate coarse boundaries of the fundamental niche, comparative analyses of closely related genomes better reveal the evolutionary imprints of past selective pressures shaping the realized niche. Our results caution against the prevailing assumption that bacteria isolated from Antarctic lakes are necessarily free-living oligotrophs, subsisting on dilute organic matter. Instead, they point to more complex life strategies involving trophic interactions and host associations, operating within the microscale heterogeneity of polar environments. With respect to cold adaptation, our findings further reinforce the view that no single genetic solution underlies survival in permanently frozen habitats. Rather, adaptation to extreme environments such as Antarctica emerges from lineage-specific combinations of gene gains and losses, superimposed—in the case of fluorescent *Pseudomonas*—on a background of intrinsic psychrotolerance. Overall, we provide an evolutionary perspective on microbial adaptation to polar environments and position AU10 as a valuable model for future experimental studies addressing the ecological and evolutionary dynamics of Antarctic bacteria.

## Data Availability

The chromosome sequence of *Pseudomonas* sp. AU10 has been deposited in GenBank under the accession number JAIUXL020000001, and its native plasmid under OQ302170. Raw sequencing reads are available in the NCBI Sequence Read Archive (SRA) under accession numbers SRR35428703 and SRR35428704. Supplementary tables (Z1–Z10) are hosted in the Zenodo repository (https://zenodo.org/records/17237991). These files include phylogenomic data, pangenome summaries, results from functional enrichment analyses, and syntenic BLASTp comparisons.

## SUPPLEMENTAL MATERIAL

Supplemental material aem.02470-25-s0001.docx Table S1 and Figures S1 to S4.

ASM does not own the copyrights to Supplemental Material that may be linked to, or accessed through, an article. The authors have granted ASM a non-exclusive, world-wide license to publish the Supplemental Material files. Please contact the corresponding author directly for reuse.

## References

[1] Palleroni NJ. 2015. *Pseudomonas*, p 1–1. *In* Bergey’s manual of systematics of archaea and bacteria. Wiley.

[2] Loper JE, Hassan KA, Mavrodi DV, Davis EW 2nd, Lim CK, Shaffer BT, Elbourne LDH, Stockwell VO, Hartney SL, Breakwell K, et al.. 2012. Comparative genomics of plant-associated Pseudomonas spp.: insights into diversity and inheritance of traits involved in multitrophic interactions. PLoS Genet 8:e1002784. doi:10.1371/journal.pgen.1002784 22792073 PMC3390384

[3] Yi B, Dalpke AH. 2022. Revisiting the intrageneric structure of the genus Pseudomonas with complete whole genome sequence information: Insights into diversity and pathogen-related genetic determinants. Infect Genet Evol 97:105183. doi:10.1016/j.meegid.2021.105183 34920102

[4] Kriss AE, Mitskevich IN, Rozanova EP, Osnitskaia LK. 1976. Microbiological studies of the Wanda Lake (Antarctica). Mikrobiologiia 45:1075–1081. 1012048

[5] Inoue K, Komagata K. 1976. Taxonomic study on obligately psychrophilic bacteria isolated from Antarctica. J Gen Appl Microbiol 22:165–176. doi:10.2323/jgam.22.165

[6] López NI, Pettinari MJ, Stackebrandt E, Tribelli PM, Põtter M, Steinbüchel A, Méndez BS. 2009. Pseudomonas extremaustralis sp. nov., a Poly(3-hydroxybutyrate) producer isolated from an antarctic environment. Curr Microbiol 59:514–519. doi:10.1007/s00284-009-9469-9 19688380

[7] Carrión O, Miñana-Galbis D, Montes MJ, Mercadé E. 2011. Pseudomonas deceptionensis sp. nov., a psychrotolerant bacterium from the Antarctic. Int J Syst Evol Microbiol 61:2401–2405. doi:10.1099/ijs.0.024919-0 21062736

[8] Kosina M, Barták M, Mašlaňová I, Pascutti AV, Sedo O, Lexa M, Sedláček I. 2013. Pseudomonas prosekii sp. nov., a novel psychrotrophic bacterium from Antarctica. Curr Microbiol 67:637–646. doi:10.1007/s00284-013-0406-6 23794042

[9] Jang GI, Lee I, Ha TT, Yoon SJ, Hwang YJ, Yi H, Yun S, Lee WS, Hwang CY. 2020. Pseudomonas neustonica sp. nov., isolated from the sea surface microlayer of the Ross Sea (Antarctica). Int J Syst Evol Microbiol 70:3832–3838. doi:10.1099/ijsem.0.004240 32511084

[10] Nováková D, Švec P, Zeman M, Busse HJ, Mašlaňová I, Pantůček R, Králová S, Krištofová L, Sedláček I. 2020. Pseudomonas leptonychotis sp. nov., isolated from Weddell seals in Antarctica. Int J Syst Evol Microbiol 70:302–308. doi:10.1099/ijsem.0.003753 31617844

[11] Nováková D, Koublová V, Sedlář K, Staňková E, Králová S, Švec P, Neumann-Schaal M, Wolf J, Koudelková S, Barták M, Sedláček I. 2023. Pseudomonas petrae sp. nov. isolated from regolith samples in Antarctica. Syst Appl Microbiol 46:126424. doi:10.1016/j.syapm.2023.126424 37167755

[12] Reddy GSN, Matsumoto GI, Schumann P, Stackebrandt E, Shivaji S. 2004. Psychrophilic pseudomonads from Antarctica: Pseudomonas antarctica sp. nov., Pseudomonas meridiana sp. nov. and Pseudomonas proteolytica sp. nov. Int J Syst Evol Microbiol 54:713–719. doi:10.1099/ijs.0.02827-0 15143013

[13] Ge HY, Zhang YH, Hu YQ, Li HR, Han W, Du Y, Hu T, Luo W, Zeng YX. 2024. Pseudomonas paeninsulae sp. nov. and Pseudomonas svalbardensis sp. nov., isolated from Antarctic intertidal sediment and Arctic soil, respectively. Int J Syst Evol Microbiol 74. doi:10.1099/ijsem.0.006466 39073408

[14] Doytchinov VV, Dimov SG. 2022. Microbial community composition of the Antarctic ecosystems: review of the bacteria, fungi, and archaea identified through an NGS-based metagenomics approach. Life (Basel) 12:916. doi:10.3390/life12060916 35743947 PMC9228076

[15] Raiger Iustman LJ, Tribelli PM, Ibarra JG, Catone MV, Solar Venero EC, López NI. 2015. Genome sequence analysis of Pseudomonas extremaustralis provides new insights into environmental adaptability and extreme conditions resistance. Extremophiles 19:207–220. doi:10.1007/s00792-014-0700-7 25316211

[16] Giovannini M, Vieri W, Bosi E, Riccardi C, Lo Giudice A, Fani R, Fondi M, Perrin E. 2024. Functional genomics of a collection of gammaproteobacteria isolated from Antarctica. Mar Drugs 22:238. doi:10.3390/md22060238 38921549 PMC11205219

[17] Moreno R, Rojo F. 2014. Features of pseudomonads growing at low temperatures: another facet of their versatility. Environ Microbiol Rep 6:417–426. doi:10.1111/1758-2229.12150 25646532

[18] Mumcu H, Sarac Cebeci ET, Menekse Kılıc M, Cebeci A, Gunes Y, Karacan I, Oztug M, Balci N, Gul Karaguler N. 2023. Identification of phenotypic and genotypic properties and cold adaptive mechanisms of novel freeze–thaw stress-resistant strain Pseudomonas mandelii from Antarctica. Polar Biol 46:169–183. doi:10.1007/s00300-023-03114-y

[19] Tribelli PM, Solar Venero EC, Ricardi MM, Gómez-Lozano M, Raiger Iustman LJ, Molin S, López NI. 2015. Novel essential role of ethanol oxidation genes at low temperature revealed by transcriptome analysis in the Antarctic bacterium Pseudomonas extremaustralis. PLoS One 10:e0145353. doi:10.1371/journal.pone.0145353 26671564 PMC4686015

[20] Benforte FC, Colonnella MA, Ricardi MM, Solar Venero EC, Lizarraga L, López NI, Tribelli PM. 2018. Novel role of the LPS core glycosyltransferase WapH for cold adaptation in the Antarctic bacterium Pseudomonas extremaustralis. PLoS One 13:e0192559. doi:10.1371/journal.pone.0192559 29415056 PMC5802925

[21] Koonin EV, Makarova KS, Wolf YI. 2021. Evolution of microbial genomics: conceptual shifts over a quarter century. Trends Microbiol 29:582–592. doi:10.1016/j.tim.2021.01.005 33541841 PMC9404256

[22] Brockhurst MA, Harrison E, Hall JPJ, Richards T, McNally A, MacLean C. 2019. The ecology and evolution of pangenomes. Curr Biol 29:R1094–R1103. doi:10.1016/j.cub.2019.08.012 31639358

[23] Shigenobu S, Watanabe H, Hattori M, Sakaki Y, Ishikawa H. 2000. Genome sequence of the endocellular bacterial symbiont of aphids Buchnera sp. APS. Nature 407:81–86. doi:10.1038/35024074 10993077

[24] Cole ST, Eiglmeier K, Parkhill J, James KD, Thomson NR, Wheeler PR, Honoré N, Garnier T, Churcher C, Harris D, et al.. 2001. Massive gene decay in the leprosy bacillus. Nature 409:1007–1011. doi:10.1038/35059006 11234002

[25] Wan JJ, Wang F, Zhang XY, Xin Y, Tian JW, Zhang YZ, Li CY, Fu HH. 2022. Genome sequencing and comparative genomics analysis of Halomonas sp. MT13 reveal genetic adaptation to deep-sea environment. Mar Genomics 61:100911. doi:10.1016/j.margen.2021.100911 35058038

[26] Lin H, Yu M, Wang X, Zhang XH. 2018. Comparative genomic analysis reveals the evolution and environmental adaptation strategies of vibrios. BMC Genomics 19:135. doi:10.1186/s12864-018-4531-2 29433445 PMC5809883

[27] Sadler MC, Wietz M, Mino S, Morris RM. 2025. Genomic diversity and adaptation in Arctic marine bacteria. mBio 16:e0155525. doi:10.1128/mbio.01555-25 40823826 PMC12421898

[28] Bosi E, Fondi M, Orlandini V, Perrin E, Maida I, de Pascale D, Tutino ML, Parrilli E, Lo Giudice A, Filloux A, Fani R. 2017. The pangenome of (Antarctic) Pseudoalteromonas bacteria: evolutionary and functional insights. BMC Genomics 18:93. doi:10.1186/s12864-016-3382-y 28095778 PMC5240218

[29] Du Y, He C, Lloyd KG, Vishnivetskaya TA, Cui H, Li B, Gong D, Fan X, Zhang D, Jiang H, Liang R. 2025. Comparative genomics reveals the high diversity and adaptation strategies of Polaromonas from polar environments. BMC Genomics 26:bmcgenomics. doi:10.1186/s12864-025-11410-6 PMC11907789 40087550

[30] Saati-Santamaría Z, Baroncelli R, Rivas R, García-Fraile P. 2022. Comparative genomics of the genus Pseudomonas reveals host- and environment-specific evolution. Microbiol Spectr 10:e0237022. doi:10.1128/spectrum.02370-22 36354324 PMC9769992

[31] Martínez-Rosales C, Castro-Sowinski S. 2011. Antarctic bacterial isolates that produce cold-active extracellular proteases at low temperature but are active and stable at high temperature. Polar Res 30:7123. doi:10.3402/polar.v30i0.7123

[32] Cagide C, Marizcurrena JJ, Vallés D, Alvarez B, Castro-Sowinski S. 2023. A bacterial cold-active dye-decolorizing peroxidase from an Antarctic Pseudomonas strain. Appl Microbiol Biotechnol 107:1707–1724. doi:10.1007/s00253-023-12405-7 36773063

[33] Fullana N, Braña V, José Marizcurrena J, Morales D, Betton J-M, Marín M, Castro-Sowinski S. 2017. Identification, recombinant production and partial biochemical characterization of an extracellular cold-active serine-metalloprotease from an Antarctic Pseudomonas isolate. AIMS Bioeng 4:386–401. doi:10.3934/bioeng.2017.3.386

[34] García-Laviña CX, Castro-Sowinski S, Ramón A. 2019. Reference genes for real-time RT-PCR expression studies in an Antarctic Pseudomonas exposed to different temperature conditions. Extremophiles 23:625–633. doi:10.1007/s00792-019-01109-4 31250110

[35] García-Laviña CX, Morel MA, García-Gabarrot G, Castro-Sowinski S. 2023. Phenotypic and resistome analysis of antibiotic and heavy metal resistance in the Antarctic bacterium Pseudomonas sp. AU10. Braz J Microbiol 54:2903–2913. doi:10.1007/s42770-023-01135-7 37783937 PMC10689667

[36] Azpiroz MF, Laviña M. 2004. Involvement of enterobactin synthesis pathway in production of microcin H47. Antimicrob Agents Chemother 48:1235–1241. doi:10.1128/AAC.48.4.1235-1241.2004 15047525 PMC375329

[37] Catalán AI, Ferreira F, Gill PR, Batista S. 2007. Production of polyhydroxyalkanoates by Herbaspirillum seropedicae grown with different sole carbon sources and on lactose when engineered to express the lacZlacY genes. Enzyme Microb Technol 40:1352–1357. doi:10.1016/j.enzmictec.2006.10.008

[38] Wick RR, Judd LM, Gorrie CL, Holt KE. 2017. Unicycler: resolving bacterial genome assemblies from short and long sequencing reads. PLoS Comput Biol 13:e1005595. doi:10.1371/journal.pcbi.1005595 28594827 PMC5481147

[39] Walker BJ, Abeel T, Shea T, Priest M, Abouelliel A, Sakthikumar S, Cuomo CA, Zeng Q, Wortman J, Young SK, Earl AM. 2014. Pilon: an integrated tool for comprehensive microbial variant detection and genome assembly improvement. PLoS One 9:e112963. doi:10.1371/journal.pone.0112963 25409509 PMC4237348

[40] Manni M, Berkeley MR, Seppey M, Simão FA, Zdobnov EM. 2021. BUSCO update: novel and streamlined workflows along with broader and deeper phylogenetic coverage for scoring of eukaryotic, prokaryotic, and viral genomes. Mol Biol Evol 38:4647–4654. doi:10.1093/molbev/msab199 34320186 PMC8476166

[41] Brettin T, Davis JJ, Disz T, Edwards RA, Gerdes S, Olsen GJ, Olson R, Overbeek R, Parrello B, Pusch GD, Shukla M, Thomason JA, Stevens R, Vonstein V, Wattam AR, Xia F. 2015. RASTtk: a modular and extensible implementation of the RAST algorithm for building custom annotation pipelines and annotating batches of genomes. Sci Rep 5:8365. doi:10.1038/srep08365 25666585 PMC4322359

[42] Olson RD, Assaf R, Brettin T, Conrad N, Cucinell C, Davis JJ, Dempsey DM, Dickerman A, Dietrich EM, Kenyon RW, et al.. 2023. Introducing the Bacterial and Viral Bioinformatics Resource Center (BV-BRC): a resource combining PATRIC, IRD and ViPR. Nucleic Acids Res 51:D678–D689. doi:10.1093/nar/gkac1003 36350631 PMC9825582

[43] Moriya Y, Itoh M, Okuda S, Yoshizawa AC, Kanehisa M. 2007. KAAS: an automatic genome annotation and pathway reconstruction server. Nucleic Acids Res 35:W182–5. doi:10.1093/nar/gkm321 17526522 PMC1933193

[44] Kanehisa M, Sato Y, Kawashima M, Furumichi M, Tanabe M. 2016. KEGG as a reference resource for gene and protein annotation. Nucleic Acids Res 44:D457–D462. doi:10.1093/nar/gkv1070 26476454 PMC4702792

[45] Caspi R, Billington R, Keseler IM, Kothari A, Krummenacker M, Midford PE, Ong WK, Paley S, Subhraveti P, Karp PD. 2020. The MetaCyc database of metabolic pathways and enzymes - a 2019 update. Nucleic Acids Res 48:D445–D453. doi:10.1093/nar/gkz862 31586394 PMC6943030

[46] Meier-Kolthoff JP, Göker M. 2019. TYGS is an automated high-throughput platform for state-of-the-art genome-based taxonomy. Nat Commun 10:2182. doi:10.1038/s41467-019-10210-3 31097708 PMC6522516

[47] Pritchard L, Glover RH, Humphris S, Elphinstone JG, Toth IK. 2016. Genomics and taxonomy in diagnostics for food security: soft-rotting enterobacterial plant pathogens. Anal Methods 8:12–24. doi:10.1039/C5AY02550H

[48] Stamatakis A. 2015. Using RAxML to infer phylogenies. Curr Protoc Bioinformatics 51:6. doi:10.1002/0471250953.bi0614s51 26334924

[49] Kumar S, Stecher G, Li M, Knyaz C, Tamura K. 2018. MEGA X: Molecular Evolutionary Genetics Analysis across computing platforms. Mol Biol Evol 35:1547–1549. doi:10.1093/molbev/msy096 29722887 PMC5967553

[50] Eren AM, Kiefl E, Shaiber A, Veseli I, Miller SE, Schechter MS, Fink I, Pan JN, Yousef M, Fogarty EC, et al.. 2021. Community-led, integrated, reproducible multi-omics with anvi’o. Nat Microbiol 6:3–6. doi:10.1038/s41564-020-00834-3 33349678 PMC8116326

[51] Shaiber A, Willis AD, Delmont TO, Roux S, Chen LX, Schmid AC, Yousef M, Watson AR, Lolans K, Esen ÖC, Lee STM, Downey N, Morrison HG, Dewhirst FE, Mark Welch JL, Eren AM. 2020. Functional and genetic markers of niche partitioning among enigmatic members of the human oral microbiome. Genome Biol 21:292. doi:10.1186/s13059-020-02195-w 33323122 PMC7739484

[52] Loperena L, Soria V, Varela H, Lupo S, Bergalli A, Guigou M, Pellegrino A, Bernardo A, Calviño A, Rivas F, Batista S. 2012. Extracellular enzymes produced by microorganisms isolated from maritime Antarctica. World J Microbiol Biotechnol 28:2249–2256. doi:10.1007/s11274-012-1032-3 22806048

[53] Schwyn B, Neilands JB. 1987. Universal chemical assay for the detection and determination of siderophores. Anal Biochem 160:47–56. doi:10.1016/0003-2697(87)90612-9 2952030

[54] King EO, Ward MK, Raney DE. 1954. Two simple media for the demonstration of pyocyanin and fluorescin. J Lab Clin Med 44:301–307. 13184240

[55] Sambrook J, Fritsch E, Maniatis T. 1989. Molecular cloning: a laboratory manual. Cold Spring Harbor Laboratory Press.

[56] Kranz RG, Gabbert KK, Madigan MT. 1997. Positive selection systems for discovery of novel polyester biosynthesis genes based on fatty acid detoxification. Appl Environ Microbiol 63:3010–3013. doi:10.1128/aem.63.8.3010-3013.1997 9251190 PMC168601

[57] Buxton R. 2016. Blood agar plates and hemolysis protocols. American Society for Microbiology:1–9.

[58] Hesse C, Schulz F, Bull CT, Shaffer BT, Yan Q, Shapiro N, Hassan KA, Varghese N, Elbourne LDH, Paulsen IT, Kyrpides N, Woyke T, Loper JE. 2018. Genome-based evolutionary history of Pseudomonas spp. Environ Microbiol 20:2142–2159. doi:10.1111/1462-2920.14130 29633519

[59] Rojo F. 2010. Carbon catabolite repression in Pseudomonas: optimizing metabolic versatility and interactions with the environment. FEMS Microbiol Rev 34:658–684. doi:10.1111/j.1574-6976.2010.00218.x 20412307

[60] Volke DC, Gurdo N, Milanesi R, Nikel PI. 2023. Time-resolved, deuterium-based fluxomics uncovers the hierarchy and dynamics of sugar processing by Pseudomonas putida. Metab Eng 79:159–172. doi:10.1016/j.ymben.2023.07.004 37454792

[61] Dvořák P, Kováč J, Lorenzo V. 2020. Biotransformation of d-xylose to d-xylonate coupled to medium-chain-length polyhydroxyalkanoate production in cellobiose-grown Pseudomonas putida EM42. Microb Biotechnol 13:1273–1283. doi:10.1111/1751-7915.13574 32363744 PMC7264884

[62] Arai H. 2011. Regulation and function of versatile aerobic and anaerobic respiratory metabolism in Pseudomonas aeruginosa. Front Microbiol 2:103. doi:10.3389/fmicb.2011.00103 21833336 PMC3153056

[63] Sherris JC, Preston NW, Shoesmith JG. 1957. The influence of oxygen and arginine on the motility of a strain of Pseudomonas sp. J Gen Microbiol 16:86–96. doi:10.1099/00221287-16-1-86 13406221

[64] Vander Wauven C, Piérard A, Kley-Raymann M, Haas D. 1984. Pseudomonas aeruginosa mutants affected in anaerobic growth on arginine: evidence for a four-gene cluster encoding the arginine deiminase pathway. J Bacteriol 160:928–934. doi:10.1128/jb.160.3.928-934.1984 6438064 PMC215798

[65] Eschbach M, Schreiber K, Trunk K, Buer J, Jahn D, Schobert M. 2004. Long-term anaerobic survival of the opportunistic pathogen Pseudomonas aeruginosa via pyruvate fermentation. J Bacteriol 186:4596–4604. doi:10.1128/JB.186.14.4596-4604.2004 15231792 PMC438635

[66] Sauer U, Eikmanns BJ. 2005. The PEP-pyruvate-oxaloacetate node as the switch point for carbon flux distribution in bacteria. FEMS Microbiol Rev 29:765–794. doi:10.1016/j.femsre.2004.11.002 16102602

[67] Dolan SK, Welch M. 2018. The Glyoxylate Shunt, 60 Years On. Annu Rev Microbiol 72:309–330. doi:10.1146/annurev-micro-090817-062257 30200852

[68] Tan IKP, Foong CP, Tan HT, Lim H, Zain NAA, Tan YC, Hoh CC, Sudesh K. 2020. Polyhydroxyalkanoate (PHA) synthase genes and PHA-associated gene clusters in Pseudomonas spp. and Janthinobacterium spp. isolated from Antarctica. J Biotechnol 313:18–28. doi:10.1016/j.jbiotec.2020.03.006 32171790

[69] Chun J, Oren A, Ventosa A, Christensen H, Arahal DR, da Costa MS, Rooney AP, Yi H, Xu X-W, De Meyer S, Trujillo ME. 2018. Proposed minimal standards for the use of genome data for the taxonomy of prokaryotes. Int J Syst Evol Microbiol 68:461–466. doi:10.1099/ijsem.0.002516 29292687

[70] Girard L, Lood C, Höfte M, Vandamme P, Rokni-Zadeh H, van Noort V, Lavigne R, De Mot R. 2021. The ever-expanding pseudomonas genus: description of 43 new species and partition of the Pseudomonas putida group. Microorganisms 9:1766. doi:10.3390/microorganisms9081766 34442845 PMC8401041

[71] Higuera-Llantén S, Pavlov MS, de Sousa LP, Vásquez-Ponce F, Parás-Silva J, Martínez JRW, Munita JM, Wozniak A, García PC, Ugalde JA, Blondel CJ, Lira F, Martínez JL, Alcalde-Rico M, Olivares-Pacheco J. 2025. Pseudomonas gelidaquae sp. nov., an Antarctic bacterium with a distinctive type III secretion system, isolated from Fildes Bay, King George Island. Antonie Van Leeuwenhoek 118:155. doi:10.1007/s10482-025-02166-w 40991091

[72] Vásquez-Ponce F, Higuera-Llantén S, Pavlov MS, Marshall SH, Olivares-Pacheco J. 2018. Phylogenetic MLSA and phenotypic analysis identification of three probable novel Pseudomonas species isolated on King George Island, South Shetland, Antarctica. Braz J Microbiol 49:695–702. doi:10.1016/j.bjm.2018.02.005 29598976 PMC6175711

[73] Marcoleta AE, Arros P, Varas MA, Costa J, Rojas-Salgado J, Berríos-Pastén C, Tapia-Fuentes S, Silva D, Fierro J, Canales N, Chávez FP, Gaete A, González M, Allende ML, Lagos R. 2022. The highly diverse Antarctic Peninsula soil microbiota as a source of novel resistance genes. Sci Total Environ 810:152003. doi:10.1016/j.scitotenv.2021.152003 34856283

[74] Lee J, Cho YJ, Yang JY, Jung YJ, Hong SG, Kim OS. 2017. Complete genome sequence of Pseudomonas antarctica PAMC 27494, a bacteriocin-producing psychrophile isolated from Antarctica. J Biotechnol 259:15–18. doi:10.1016/j.jbiotec.2017.08.013 28818601

[75] Dharmaprakash A, Reghunathan D, Sivakumar KC, Prasannakumar M, Thomas S. 2016. Insights into the genome sequences of an N-Acyl homoserine lactone molecule producing two Pseudomonas spp. isolated from the Arctic. Genome Announc 4:767–783. doi:10.1128/genomeA.00767-16 PMC4974316 27491995

[76] Ramón A, Esteves A, Villadóniga C, Chalar C, Castro-Sowinski S. 2023. A general overview of the multifactorial adaptation to cold: biochemical mechanisms and strategies. Braz J Microbiol 54:2259–2287. doi:10.1007/s42770-023-01057-4 37477802 PMC10484896

[77] Kamal SM, Rybtke ML, Nimtz M, Sperlein S, Giske C, Trček J, Deschamps J, Briandet R, Dini L, Jänsch L, Tolker-Nielsen T, Lee C, Römling U. 2019. Two FtsH proteases contribute to fitness and adaptation of Pseudomonas aeruginosa clone C strains. Front Microbiol 10:1372. doi:10.3389/fmicb.2019.01372 31338071 PMC6629908

[78] Vacheron J, Péchy-Tarr M, Brochet S, Heiman CM, Stojiljkovic M, Maurhofer M, Keel C. 2019. T6SS contributes to gut microbiome invasion and killing of an herbivorous pest insect by plant-beneficial Pseudomonas protegens. ISME J 13:1318–1329. doi:10.1038/s41396-019-0353-8 30683920 PMC6474223

[79] Rangel LI, Henkels MD, Shaffer BT, Walker FL, Davis EW, Stockwell VO, Bruck D, Taylor BJ, Loper JE. 2016. Characterization of toxin complex gene clusters and insect toxicity of bacteria representing four subgroups of Pseudomonas fluorescens. PLoS One 11:e0161120. doi:10.1371/journal.pone.0161120 27580176 PMC5006985

[80] Meusch D, Gatsogiannis C, Efremov RG, Lang AE, Hofnagel O, Vetter IR, Aktories K, Raunser S. 2014. Mechanism of Tc toxin action revealed in molecular detail. Nature 508:61–65. doi:10.1038/nature13015 24572368

[81] Green ER, Mecsas J. 2016. Bacterial secretion systems: an overview. Microbiol Spectr 4:213–239. doi:10.1128/microbiolspec.VMBF-0012-2015 PMC4804464 26999395

[82] Graña-Miraglia L, Geney Higuita JL, Salazar JC, Guaya Iñiguez D, Alcolado León C, García-Angulo VA. 2024. Total substitution and partial modification of the set of non-ribosomal peptide synthetases clusters lead to pyoverdine diversity in the Pseudomonas fluorescens complex. Front Microbiol 15:1421749. doi:10.3389/fmicb.2024.1421749 39224222 PMC11366639

[83] Damron FH, Oglesby-Sherrouse AG, Wilks A, Barbier M. 2016. Dual-seq transcriptomics reveals the battle for iron during Pseudomonas aeruginosa acute murine pneumonia. Sci Rep 6:39172. doi:10.1038/srep39172 27982111 PMC5159919

[84] Lam JS, Taylor VL, Islam ST, Hao Y, Kocíncová D. 2011. Genetic and functional diversity of Pseudomonas aeruginosa lipopolysaccharide. Front Microbiol 2:118. doi:10.3389/fmicb.2011.00118 21687428 PMC3108286

[85] Kupferschmied P, Chai T, Flury P, Blom J, Smits THM, Maurhofer M, Keel C. 2016. Specific surface glycan decorations enable antimicrobial peptide resistance in plant-beneficial pseudomonads with insect-pathogenic properties. Environ Microbiol 18:4265–4281. doi:10.1111/1462-2920.13571 27727519

[86] Raymond CK, Sims EH, Kas A, Spencer DH, Kutyavin TV, Ivey RG, Zhou Y, Kaul R, Clendenning JB, Olson MV. 2002. Genetic variation at the O-antigen biosynthetic locus in Pseudomonas aeruginosa. J Bacteriol 184:3614–3622. doi:10.1128/JB.184.13.3614-3622.2002 12057956 PMC135118

[87] Jayaraman J, Jones WT, Harvey D, Hemara LM, McCann HC, Yoon M, Warring SL, Fineran PC, Mesarich CH, Templeton MD. 2020. Variation at the common polysaccharide antigen locus drives lipopolysaccharide diversity within the Pseudomonas syringae species complex. Environ Microbiol 22:5356–5372. doi:10.1111/1462-2920.15250 32985740 PMC7820976

[88] Hu YQ, Zeng YX, Du Y, Zhao W, Li HR, Han W, Hu T, Luo W. 2023. Comparative genomic analysis of two Arctic Pseudomonas strains reveals insights into the aerobic denitrification in cold environments. BMC Genomics 24:534. doi:10.1186/s12864-023-09638-1 37697269 PMC10494350

[89] Raposo A, Pérez E, Faria CT, Ferrús MA, Carrascosa C. 2017. Food spoilage by *Pseudomonas* spp.-An overview, p 41–71. *In* Foodborne pathogens and antibiotic resistance. Wiley.

[90] Mykytczuk NCS, Foote SJ, Omelon CR, Southam G, Greer CW, Whyte LG. 2013. Bacterial growth at -15°C; molecular insights from the permafrost bacterium Planococcus halocryophilus Or1. ISME J 7:1211–1226. doi:10.1038/ismej.2013.8 23389107 PMC3660685

[91] de Wit R, Bouvier T. 2006. “Everything is everywhere, but, the environment selects”; what did Baas Becking and Beijerinck really say? Environ Microbiol 8:755–758. doi:10.1111/j.1462-2920.2006.01017.x 16584487

[92] Martiny JBH, Bohannan BJM, Brown JH, Colwell RK, Fuhrman JA, Green JL, Horner-Devine MC, Kane M, Krumins JA, Kuske CR, Morin PJ, Naeem S, Ovreås L, Reysenbach A-L, Smith VH, Staley JT. 2006. Microbial biogeography: putting microorganisms on the map. Nat Rev Microbiol 4:102–112. doi:10.1038/nrmicro1341 16415926

[93] Hanson CA, Fuhrman JA, Horner-Devine MC, Martiny JBH. 2012. Beyond biogeographic patterns: processes shaping the microbial landscape. Nat Rev Microbiol 10:497–506. doi:10.1038/nrmicro2795 22580365

[94] Methé BA, Nelson KE, Deming JW, Momen B, Melamud E, Zhang X, Moult J, Madupu R, Nelson WC, Dodson RJ, et al.. 2005. The psychrophilic lifestyle as revealed by the genome sequence of Colwellia psychrerythraea 34H through genomic and proteomic analyses. Proc Natl Acad Sci USA 102:10913–10918. doi:10.1073/pnas.0504766102 16043709 PMC1180510

[95] Tribelli PM, López NI. 2018. Reporting key features in cold-adapted bacteria. Life (Basel) 8:8. doi:10.3390/life8010008 29534000 PMC5871940

[96] Jo J, Cortez KL, Cornell WC, Price-Whelan A, Dietrich LEP. 2017. An orphan cbb3-type cytochrome oxidase subunit supports Pseudomonas aeruginosa biofilm growth and virulence. eLife 6. doi:10.7554/eLife.30205 PMC5697931 29160206

[97] Butaitė E, Baumgartner M, Wyder S, Kümmerli R. 2017. Siderophore cheating and cheating resistance shape competition for iron in soil and freshwater Pseudomonas communities. Nat Commun 8:414. doi:10.1038/s41467-017-00509-4 28871205 PMC5583256

[98] Hirai T, Osamura T, Ishii M, Arai H. 2016. Expression of multiple cbb_3_ cytochrome c oxidase isoforms by combinations of multiple isosubunits in Pseudomonas aeruginosa. Proc Natl Acad Sci USA 113:12815–12819. doi:10.1073/pnas.1613308113 27791152 PMC5111723

[99] Krajewski SS, Nagel M, Narberhaus F. 2013. Short ROSE-like RNA thermometers control IbpA synthesis in Pseudomonas species. PLoS One 8:e65168. doi:10.1371/journal.pone.0065168 23741480 PMC3669281

[100] Peng W, Wang Y, Fu Y, Deng Z, Lin S, Liang R. 2022. Characterization of the tellurite-resistance properties and identification of the core function genes for tellurite resistance in Pseudomonas citronellolis SJTE-3. Microorganisms 10:95. doi:10.3390/microorganisms10010095 35056544 PMC8779313

[101] Sundin GW, Kidambi SP, Ullrich M, Bender CL. 1996. Resistance to ultraviolet light in Pseudomonas syringae: sequence and functional analysis of the plasmid-encoded rulAB genes. Gene 177:77–81. doi:10.1016/0378-1119(96)00273-9 8921848

[102] Cordero RR, Feron S, Damiani A, Redondas A, Carrasco J, Sepúlveda E, Jorquera J, Fernandoy F, Llanillo P, Rowe PM, Seckmeyer G. 2022. Persistent extreme ultraviolet irradiance in Antarctica despite the ozone recovery onset. Sci Rep 12:1266. doi:10.1038/s41598-022-05449-8 35075240 PMC8786956

[103] Kulikov EE, Majewska J, Prokhorov NS, Golomidova AK, Tatarskiy EV, Letarov AV. 2017. Effect of O-acetylation of O antigen of Escherichia coli lipopolysaccharide on the nonspecific barrier function of the outer membrane. Microbiology (Reading, Engl) 86:310–316. doi:10.1134/S0026261717030080

[104] Rodriguez-Valera F, Martin-Cuadrado AB, López-Pérez M. 2016. Flexible genomic islands as drivers of genome evolution. Curr Opin Microbiol 31:154–160. doi:10.1016/j.mib.2016.03.014 27085300

[105] Blackburn M, Golubeva E, Bowen D, Ffrench-Constant RH. 1998. A novel insecticidal toxin from photorhabdus luminescens, toxin complex a (Tca), and its histopathological effects on the midgut of Manduca sexta. Appl Environ Microbiol 64:3036–3041. doi:10.1128/AEM.64.8.3036-3041.1998 9687470 PMC106812

[106] Brugirard-Ricaud K, Duchaud E, Givaudan A, Girard PA, Kunst F, Boemare N, Brehélin M, Zumbihl R. 2005. Site-specific antiphagocytic function of the Photorhabdus luminescens type III secretion system during insect colonization. Cell Microbiol 7:363–371. doi:10.1111/j.1462-5822.2004.00466.x 15679839

[107] Kozeretska I, Serga S, Kovalenko P, Gorobchyshyn V, Convey P. 2022. Belgica antarctica (Diptera: Chironomidae): a natural model organism for extreme environments. Insect Sci 29:2–20. doi:10.1111/1744-7917.12925 33913258

[108] Janiec K. 1996. The comparison of freshwater invertebrates of Spitsbergen (Arctic) and King George Island (Antarctic). Pol Polar Res 17:173–202.

[109] Morel MA, Braña V, Martínez-Rosales C, Cagide C, Castro-Sowinski S. 2015. Five-year bio-monitoring of aquatic ecosystems near Artigas Antarctic scientific base, King George Island. Adv Polar Sci 26:102–106. doi:10.13679/j.advps.2015.1.00102

