# Erratum for García-Laviña et al., “From genome to lifestyle: adaptive strategies of *Pseudomonas* sp. AU10 and its Antarctic lineage”

**DOI:** 10.1128/aem.01185-26

**Published:** 2026-07-23

**Authors:** César X. García-Laviña, Pablo Smircich, Susana Castro-Sowinski

## ERRATUM

Volume 92, no. 4, e02470-25, 2026, https://doi.org/10.1128/aem.02470-25. Discussion, 6th paragraph: “to avoid siderophore piracy (98)” should read ““to avoid siderophore piracy (97).”

Discussion, 6th paragraph: “in *Pseudomonas citronellolis* (99)” should read “in *Pseudomonas citronellolis* (98).”

Discussion, 7th paragraph: “UV-resistant phenotypes (97)” should read “UV-resistant phenotypes (99).”

References: References 86 to 103 were misnumbered in the original References list; the correct numbering is as follows.

## REFERENCES

**86.** Hu YQ, Zeng YX, Du Y, Zhao W, Li HR, Han W, Hu T, Luo W. 2023. Comparative genomic analysis of two Arctic *Pseudomonas* strains reveals insights into the aerobic denitrification in cold environments. BMC Genomics 24:534. https://doi.org/10.1186/s12864-023-09638-1

**87.** Raposo A, Pérez E, Faria CT, Ferrús MA, Carrascosa C. 2017. Food spoilage by *Pseudomonas* spp.-An overview, p 41-71. In Foodborne pathogens and antibiotic resistance. Wiley.

**88.** Mykytczuk NCS, Foote SJ, Omelon CR, Southam G, Greer CW, Whyte LG. 2013. Bacterial growth at -15°C; molecular insights from the permafrost bacterium *Planococcus halocryophilus* Or1. ISME J 7:1211-1226. https://doi.org/10.1038/ismej.2013.8

**89.** de Wit R, Bouvier T. 2006. “Everything is everywhere, but, the environment selects”; what did Baas Becking and Beijerinck really say? Environ Microbiol 8:755-758. https://doi.org/10.1111/j.1462-2920.2006.01017.x

**90.** Martiny JBH, Bohannan BJM, Brown JH, Colwell RK, Fuhrman JA, Green JL, Horner-Devine MC, Kane M, Krumins JA, Kuske CR, Morin PJ, Naeem S, Ovreås L, Reysenbach A-L, Smith VH, Staley JT. 2006. Microbial biogeography: putting microorganisms on the map. Nat Rev Microbiol 4:102-112. https://doi.org/10.1038/nrmicro1341

**91.** Hanson CA, Fuhrman JA, Horner-Devine MC, Martiny JBH. 2012. Beyond biogeographic patterns: processes shaping the microbial landscape. Nat Rev Microbiol 10:497-506. https://doi.org/10.1038/nrmicro2795

**92.** Methé BA, Nelson KE, Deming JW, Momen B, Melamud E, Zhang X, Moult J, Madupu R, Nelson WC, Dodson RJ, et al. 2005. The psychrophilic lifestyle as revealed by the genome sequence of *Colwellia psychrerythraea* 34H through genomic and proteomic analyses. Proc Natl Acad Sci USA 102:10913-10918. https://doi.org/10.1073/pnas.0504766102

**93.** Tribelli PM, López NI. 2018. Reporting key features in cold-adapted bacteria. Life (Basel) 8:8. https://doi.org/10.3390/life8010008

**94.** Jo J, Cortez KL, Cornell WC, Price-Whelan A, Dietrich LEP. 2017. An orphan cbb3-type cytochrome oxidase subunit supports *Pseudomonas aeruginosa* biofilm growth and virulence. eLife 6. https://doi.org/10.7554/eLife.30205

**95.** Hirai T, Osamura T, Ishii M, Arai H. 2016. Expression of multiple cbb3 cytochrome c oxidase isoforms by combinations of multiple isosubunits in *Pseudomonas aeruginosa*. Proc Natl Acad Sci USA 113:12815-12819. https://doi.org/10.1073/pnas.1613308113

**96.** Krajewski SS, Nagel M, Narberhaus F. 2013. Short ROSE-like RNA thermometers control IbpA synthesis in *Pseudomonas* species. PLoS One 8:e65168. https://doi.org/10.1371/journal.pone.0065168

**97.** Butaitė E, Baumgartner M, Wyder S, Kümmerli R. 2017. Siderophore cheating and cheating resistance shape competition for iron in soil and freshwater *Pseudomonas* communities. Nat Commun 8:414. https://doi.org/10.1038/s41467-017-00509-4

**98.** Peng W, Wang Y, Fu Y, Deng Z, Lin S, Liang R. 2022. Characterization of the tellurite-resistance properties and identification of the core function genes for tellurite resistance in *Pseudomonas citronellolis* SJTE-3. Microorganisms 10:95. https://doi.org/10.3390/microorganisms10010095

**99.** Sundin GW, Kidambi SP, Ullrich M, Bender CL. 1996. Resistance to ultraviolet light in *Pseudomonas syringae*: sequence and functional analysis of the plasmid-encoded rulAB genes. Gene 177:77-81. https://doi.org/10.1016/0378-1119(96)00273-9

**100.** Cordero RR, Feron S, Damiani A, Redondas A, Carrasco J, Sepúlveda E, Jorquera J, Fernandoy F, Llanillo P, Rowe PM, Seckmeyer G. 2022. Persistent extreme ultraviolet irradiance in Antarctica despite the ozone recovery onset. Sci Rep 12:1266. https://doi.org/10.1038/s41598-022-05449-8

**101.** Kulikov EE, Majewska J, Prokhorov NS, Golomidova AK, Tatarskiy EV, Letarov AV. 2017. Effect of O-acetylation of O antigen of *Escherichia coli* lipopolysaccharide on the nonspecific barrier function of the outer membrane. Microbiology (Reading, Engl) 86:310-316. https://doi.org/10.1134/S0026261717030080

**102.** Raymond CK, Sims EH, Kas A, Spencer DH, Kutyavin TV, Ivey RG, Zhou Y, Kaul R, Clendenning JB, Olson MV. 2002. Genetic variation at the O-antigen biosynthetic locus in *Pseudomonas aeruginosa*. J Bacteriol 184:3614-3622. https://doi.org/10.1128/JB.184.13.3614-3622.2002

**103.** Jayaraman J, Jones WT, Harvey D, Hemara LM, McCann HC, Yoon M, Warring SL, Fineran PC, Mesarich CH, Templeton MD. 2020. Variation at the common polysaccharide antigen locus drives lipopolysaccharide diversity within the *Pseudomonas syringae* species complex. Environ Microbiol 22:5356-5372. https://doi.org/10.1111/1462-2920.15250

