# Supplementary material for: From genome to lifestyle: adaptive strategies of Pseudomonas sp. AU10 and its Antarctic lineage
Source: Appl Environ Microbiol. 2026 Mar 24;92(4):e02470-25. doi: 10.1128/aem.02470-25 (PMC13101481; doi:10.1128/aem.02470-25)
Supplement: Supplemental material — Table S1 and Figures S1 to S4. [file aem.02470-25-s0001.docx]

**Supplementary material**

| Table S1. Biochemical tests performed on *Pseudomonas* sp. AU10   \| Biochemical test \| Positive result \| Negative result \| \| --- \| --- \| --- \| \| Specific enzymatic tests \| oxidase, catalase, phosphatase^2^, arginine deiminase^1^, lysine decarboxylase^2^, ornithine decarboxylase^3^, proline peptidase^2^, γ-glutamil transpeptidase^2^, urease^2 3^, PYR^3^ \| β-galactosidase^1 2 3^, α-galactosidase^3^, α and β-glucosidase^2^, xylanase^2^, β-xylosidase^3^, α-arabinofuranosidase^2^, β-glucuronidase^2 3^, β-N-acetyl-glucosaminidase^2^, phosphodiesterase^2^, phospholipase D^2^, phenylalanine deaminase^3^, tryptophan deaminase^1^, tryptophanase (indole)^1 3^, gelatinase^1^ \| \| Oxidation of carbon sources \| glycine^2^, citrate^1 2 3^, malonate^2 3^ \| - \| \| Acid production \| glucose^1 3^, mannose^2 3^, galactose^2 3^, arabinose^2 3^, xylose^3^ \| lactose^3^, maltose^3^, fructose^3^, raffinose^3^, trehalose^3^, sucrose^1 2 3^, rhamnose^1 2 3^, cellobiose^3^, melibiose^2 3^, melezitose^3^, inulin^3^, gluconate^3^, glycerol^3^, dulcitol^3^, inositol^2 3^, sorbitol^1 2 3^, mannitol^1 2 3^, adonitol^1 2 3^, arabitol^3^, erythritol^3^, xylitol^3^, sorbose^3^, salicin^3^, amygdalin^1^ \| \| Other assays \| tetrazolium reduction^2^ \| esculin^2 3^, H_2_S production^1 3^, Voges-Proskauer^1^, Methyl red^3^, nitrate reduction^1 3^, N_2_ production^1^ \|   1 Results obtained with API 20E (BioMérieux)  2 Results obtained with BD BBL Crystal Enteric/Nonfermenter ID kit  3 Results obtained with HiMedia kits (KB002, KB009, KB016) |
| --- | --- | --- | --- | --- | --- | --- | --- | --- | --- | --- | --- | --- | --- | --- | --- |

| 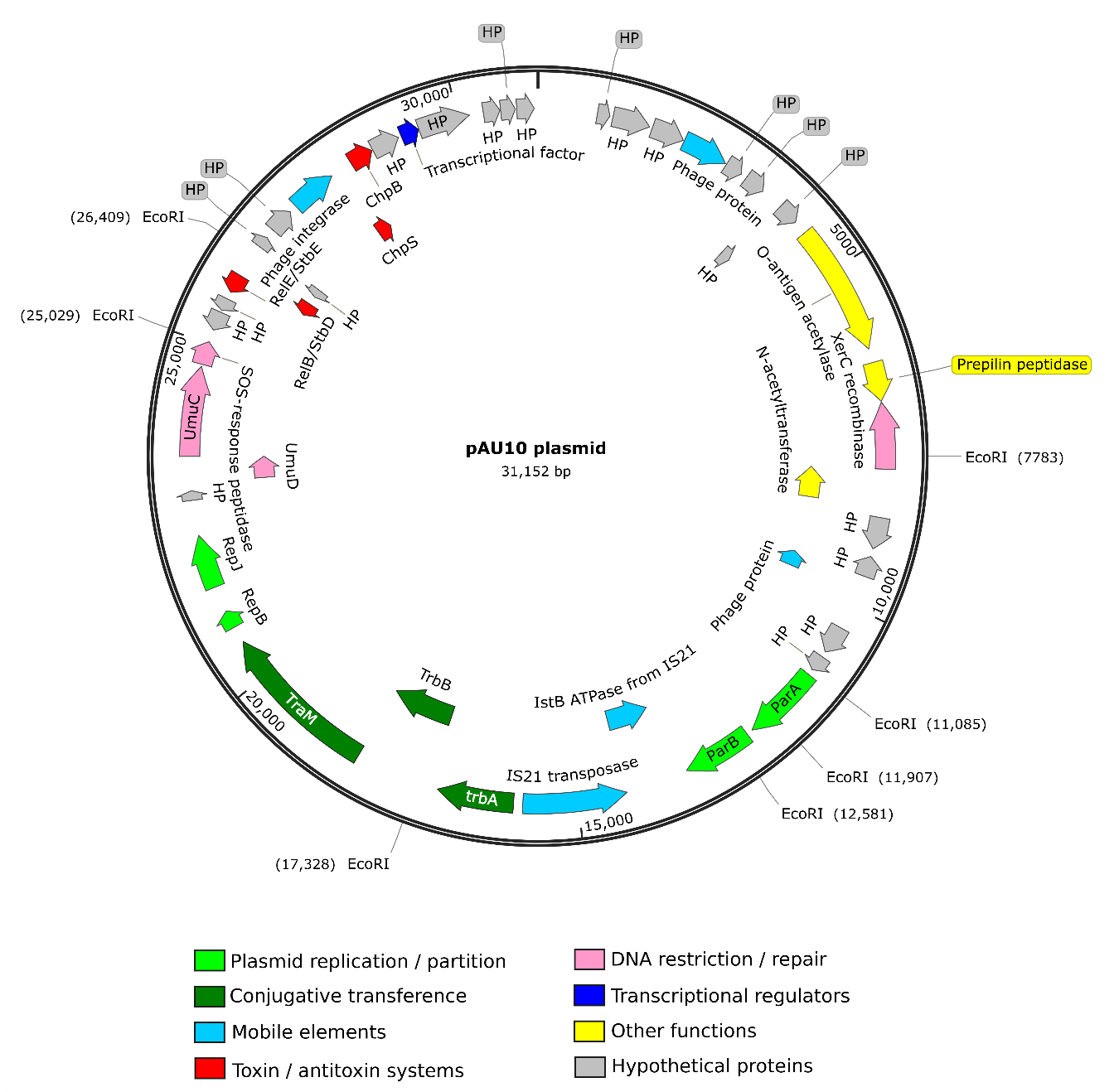  Figure S1. Circular map of *Pseudomonas* sp. AU10 natural plasmid pAU10. The identified genes were colored according to their predicted function. |
| --- |

| 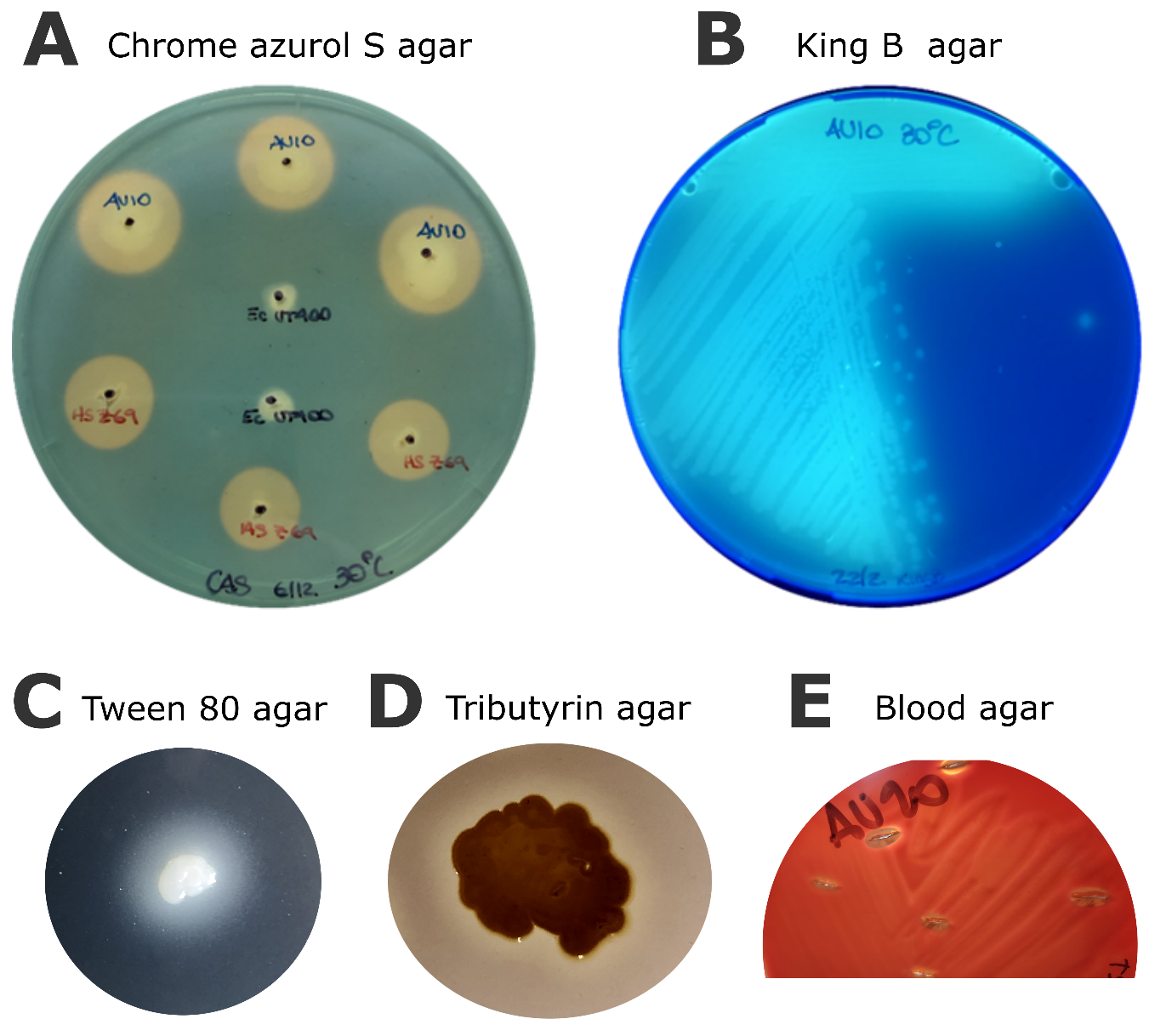  Figure S2. Plate assays performed with *Pseudomonas* sp. AU10. (A) Siderophore production on CAS medium, using *H. seropedicae* Z69 as positive control and the enterobactin-deficient strain *E. coli* UT400 as negative control (see Bacterial strains and growth conditions in Materials and Methods). (B) Fluorescence emission due to pyoverdine production on King B medium when exposed to UV light. (C) Lipase activity (see opalescent zone around spot culture). (D) Esterase activity (see clearance halo around spot culture stained with lugol). (E) β-hemolysis of AU10 culture on stabbed agar. |
| --- |


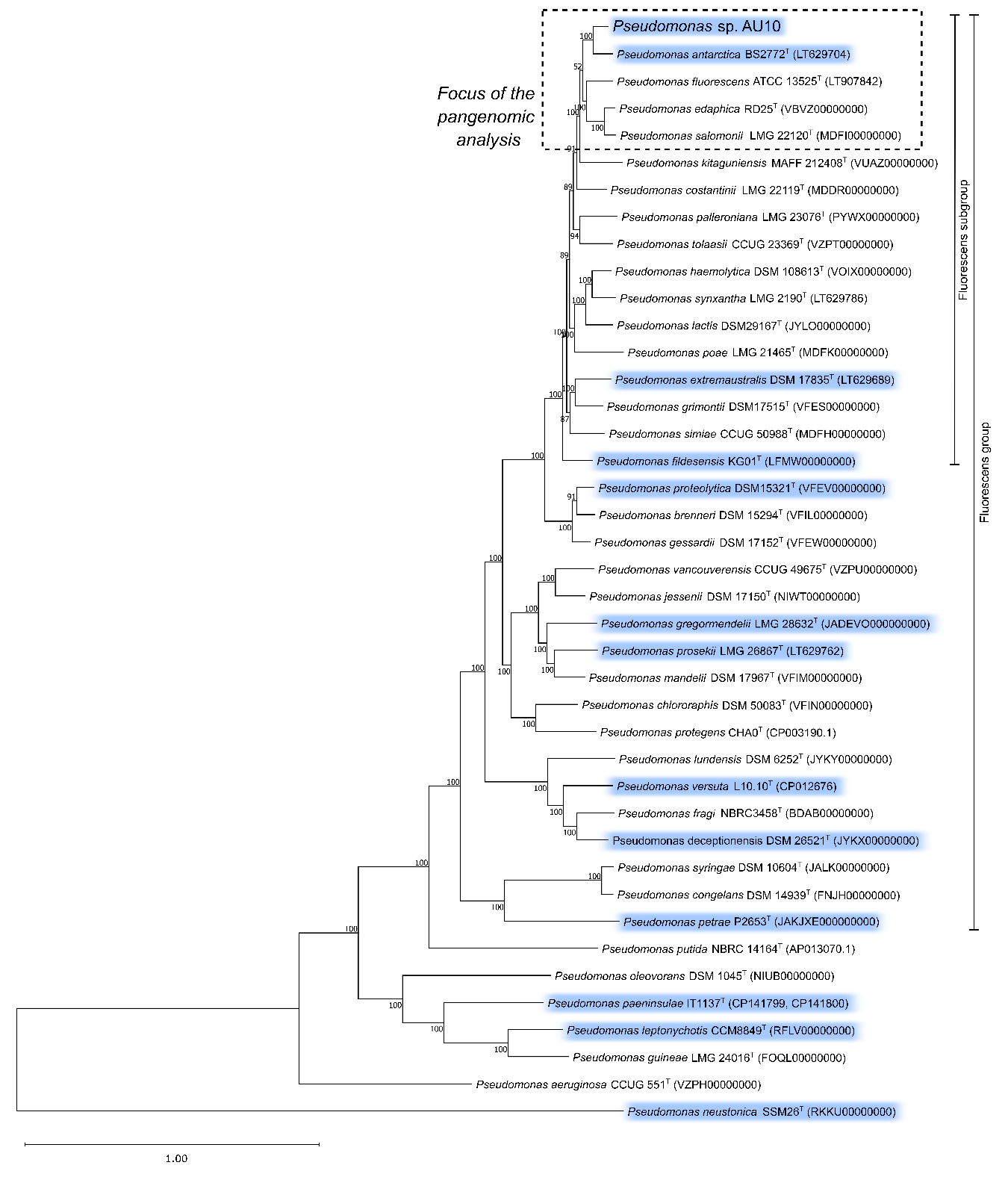


**Figure S3.** Phylogenomic tree of *Pseudomonas* sp. AU10 and 40 type strains from the genus. The maximum-likelihood tree was generated from 599 shared single-copy orthologous genes. Nodes were statistically supported through 100 rounds of bootstrap. Type strains of Antarctic origin are highlighted with a blue background. The dashed box encloses the species later selected for more detailed analyses in this work (Figure 2, phylogenomic analysis; Figure 3, pangenomic analysis).


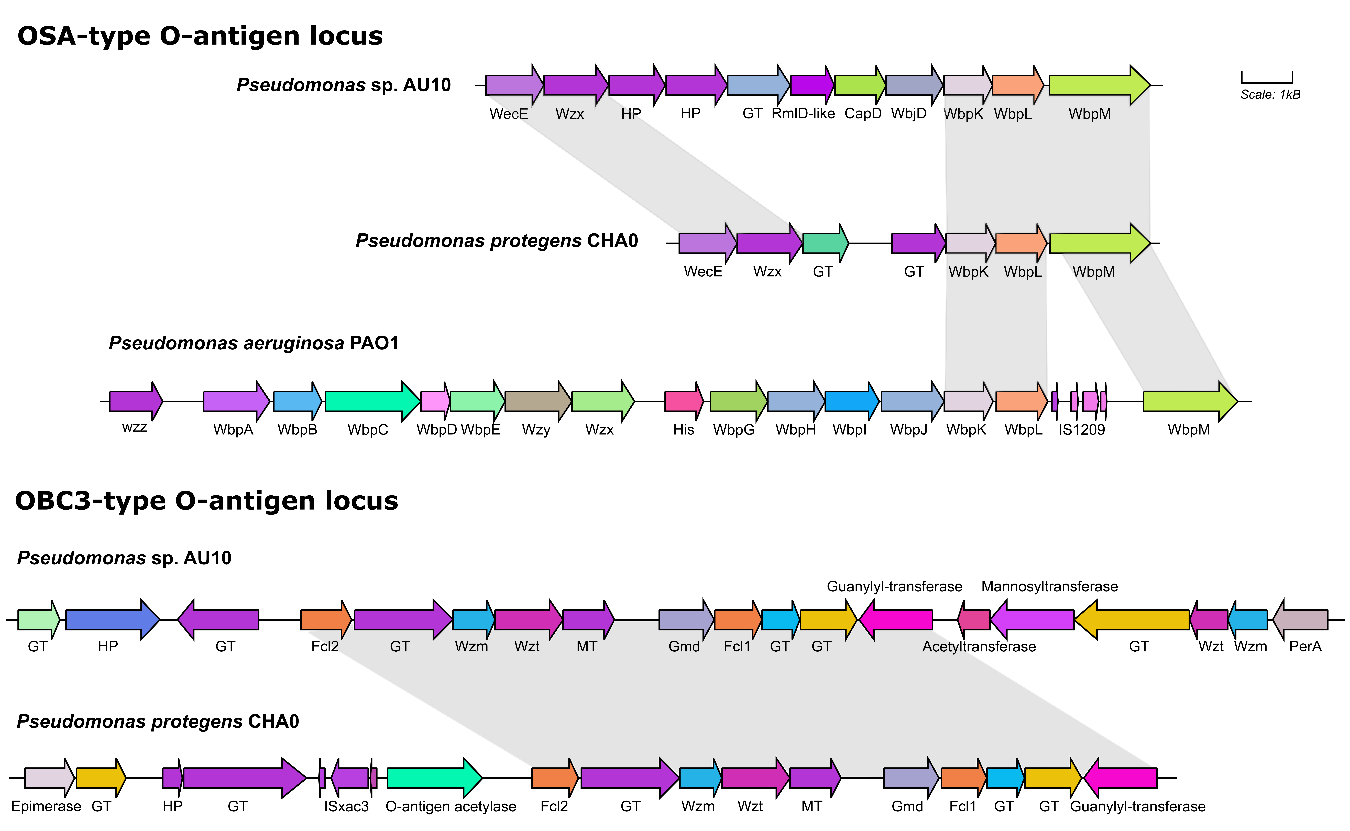


**Figure S4.** Gene clusters involved in lipopolysaccharide (LPS) O-antigen synthesis in AU10 and related strains. Regions of homology are shown in grey. Abbreviations: GT, glycosyltransferase; MT, methyltransferase; Wzx, OSA flipase; Wzy, OSA polymerase; Wzm and Wzt, OBC3 ABC transporter components; Fcl, GDP-L-fucose synthase; WecE, dTDP-4-amino-4,6-dideoxygalactose transaminase; CapD, galactowaldenase; PerA, GDP-perosamine synthase; HP, hypothetical protein.
